# Cosine similarity measures between q-rung orthopair linguistic sets and their application to group decision making problems

**DOI:** 10.1038/s41598-022-18694-8

**Published:** 2022-08-24

**Authors:** Jawad Ali, Muhammad Naeem

**Affiliations:** 1 Institute of Numerical Sciences, Kohat University of Science and Technology, Kohat, KPK Pakistan; 2 Umm Al-Qura University, Makkah, Saudi Arabia

**Keywords:** Engineering, Mathematics and computing

## Abstract

The q-rung orthopair linguistic set (q-ROLS), a combined version of linguistic term sets and q-rung orthopair fuzzy set, is an efficient mathematical tool to accomplish the imprecise information while solving the decision-making problems. Under this environment, we propose additional operations and relations to deal with the decision information, and some properties are well proved. Furthermore, we propound some cosine similarity measures and weighted cosine similarity measures for q-ROLSs based on the traditional cosine similarity measures with a brief study of related properties. In the proposed similarity measures, various linguistic scale functions are utilized in order to take into account the semantics of linguistic terms. Besides this, we employ the stated q-rung orthopair linguistic similarity measures to multi-criteria group decision making problems, in which the weights of DMs are delineated by the projection of individual decisions on the ideal decision results. At last, a numerical example is used to demonstrate the practicality of the suggested technique, and its efficacy is validated by comparison with prevailing techniques. The suggested method’s sensitivity and stability analyses are also provided.

## Introduction

Decision making procedure involves examining a limited set of choices and identifying them with a certain degree of confidence for decision makers (DMs) when all the rules are thought out simultaneously. All choices include both accurate information and subjective data from experts. In general, however, information collected from various data sources is limited and subject to significant uncertainty. To deal with such uncertainties, the idea of fuzzy set (FS), pioneered by Zadeh^1^ offers a prominent device to cope with unsure and unpredictable facts within the surroundings of actual existence issues. In FS, each element contains the membership function, whose range lies in [0,1]. Since its foundation, researchers have paid great attention to them and applied them in various fields, but its scope is limited because it only considers each element’s membership grade and does not contain its non-membership grade. To address this issue, Atanassov^2^ established the intuitionistic fuzzy set (IFS) by incorporating the non-membership grade of an element to the FS with the condition that the sum of the membership and non-membership grades should be at most one. With the aid of IFS, DMs are able to handle complex and challenging problems with ease. An increasing number of researchers have begun to focus on the IFS since its introduction. However, it is still noticed that the feasible region of IFS is a triangular region and is limited in access. For instance, when a DM states information such as (0.5, 0.6), where the membership and non-membership grades have the condition $0.5+0.6=1.1>1$, and so in such cases, IFS does not effectively cope with it.

To address the situations mentioned above, Yager^3^ put forward the notion of Pythagorean fuzzy set (PFS) as a generalization of IFS whose prominent characteristic is that the sum of the square of membership and non-membership grades is less than or equal to one. From its structure, it is clear that the searching space of this set is broader than the FS and IFS. To study the influence of PFS on decision making, numerous researchers have proposed different types of algorithms. For example, Garg^4^ studied correlation coefficients based on PFS pairs. Peng and Yang^5^ discussed several types of PFS baseline results. Onar et al.^6^ presented technique of order preference similarity to the ideal solution (TOPSIS) in a PFS environment for solving decision ranking problems. Garg^7^ propound weighted aggregation operators for PFS. Khan et al.^8^ improved the existing VIKOR method by defining a novel dissimilarity measure for PFS. Peng and Selvachandran^9^ reviewed various decision making approaches to solve the problems under the Pythagorean fuzzy environment.

More recently, Yager^10^ came up with the notion of q-rung otyhopair fuzzy set (q-ROFS), in which the sum of the *q*th power of the membership and non-membership grades is at most 1. Obviously, q-ROFS extends the existing IFS and PFS and provides the DMs with more freedom to express their assessment information. Liu and Wang^11^ established q-rung orthopair fuzzy weighted averaging and geometric operators with a brief study of their characteristics. Wei et al.^12^ presented a series of q-rung orthopair fuzzy Maclaurin symmetric mean (MSM) operators and their implication to decision making problems. Peng et al.^13^ defined score function-based distance measures for dealing with comparison problems under q-rung orthopair fuzzy setting. Pinar and Boran^14^ discussed supplier selection problem based on their proposed novel distance measure under the background of q-ROFS. Liu et al.^15^ introduced Power MSM operator and listed its required properties with detailed proof. Based on the proposed operator, they further investigated the multi-criteria group decision making (MCGDM) approach and discussed its validity. Rani and Mishra^16^ studied weighted aggregated sum product assessment (WASPAS) methodology based on their proposed score function and similarity measures to address the alternative-fuel technology selection problem within q-ROFS context. Ali^17^ pointed out the drawbacks of the existing q-rung orthopair fuzzy score functions and defined a novel score function. Based on the proposed novel score function, he extended the measurement alternatives and ranking according to the compromise solution (MARCOS) method and discussed its application to solid waste management. Verma^18^ explored order-$\alpha$ divergence and entropy measures for q-ROFSs and addressed the best enterprise resource planning system selection problem to illustrate their applicability. The authors of Ref.^19^ critically analyzed the existing attempts of q-rung orthopair fuzzy divergence measure and formulated an improved divergence measure. Based on the proposed measure, they extended multi-attribute border approximation area comparison decision method for selection problems. Khan et al.^20^ examined the existing ranking strategies for q-rung orthopair fuzzy values and proposed a novel graphical ranking method based on the hesitancy index and entropy. Peng and Liu^21^ gave some formulas for information measures of q-ROFSs and employed them to pattern recognition and medical diagnosis. The similarity measures of q-ROFSs based on cosine function were expounded by Wang et al.^22^. To fill the gaps of the existing distance measures of q-rung picture fuzzy sets, in Ref.^23^, authors devised a novel distance measure along with detail proof of its required properties.

There are some situations wherein DMs opt to make qualitative decisions rather than quantitative decisions because of a loss of time and expertise. Zadeh’s^24^ linguistic variables are powerful equipment to make qualitative selections. However, traditional linguistic variables can only reflect the qualitative preferences of DMs, and the grade of membership or non-membership of an element of a certain concept is ignored. Therefore, Wang and Li^25^ dispatched the idea of intuitionistic linguistic set (ILS), by connecting LTS with IFS. Based on the proposed induced aggregation operators, Liu et al.^26^ investigated the MCGDM technique under intuitionistic linguistic environment and analyzed possibilities for its use in low carbon supplier selection. Yu et al.^27^ studied an interactive MCGDM methodology based on nonlinear programming and an acronym in Portuguese of interactive and multicriteria decision making (TODIM) method using intuitionistic linguistic data. A novel dempster-shafer evidence theory (DSET)-based MCGDM algorithm with intuitionistic linguistic data was given by^28^. In view of the significance and the ordered position of the input arguments, Chen et al.^29^ defined an intuitionistic linguistic hybrid weighted logarithmic averaging distance (ILHWLAD) operator and provided the decision making methodology based on the ILHWLAD to address the finest concrete selection problem. Though ILSs are widely studied and used, their ability to describe fuzzy information is limited. Motivated by the power of q-ROFS, Li et al.^30^ further extended ILS to q-rung orthopair linguistic set (q-ROLS). They established a family of Heronian mean operators under a q-rung orthopair linguistic setting and demonstrated their applications in MCGDM problems. It is evident that q-ROLS is more general than ILS due to the fact that ILS is the special case of q-ROLS when q = 1. It is worth noting that the space of acceptable information can increase with the rung q increases, and more uncertainty information satisfy the bounding constraint. Therefore, q-ROLS is more suitable to describe fuzzy information than ILS. So far, very limited work has been done over q-ROLS, and there is capacity of much work to expand its scope of applications.

The idea of projection was initially projected within the computational work of physics. Currently, it’s become a significant tool for handling MCGDM problems. The projection model shows both distance and angle between two alternatives, and it can reflect the distance more accurately than other distance measures. Xu and Hu^31^ detailed two novel projection models to respectively depict the similarity between the alternative and ideal solution under intuitionistic fuzzy setting. Liang et al.^32^ conferred and employed the Pythagorean fuzzy projection model to resolve pythagorean fuzzy MCGDM problems. A projection technique with integrating Linguistic Z-numbers become defined through Huang et al.^33^ for comparing the reliability and protection of aircraft landing machines. Yue^34^ highlighted some shortcomings of the original projection computation method and studied two normalized projection models to overcome the defects in solving MCGDM problem. Liu and You^35^ propound a bidirectional projection measure for linguistic neutrosophic setting. In addition, the projection method can also be combined with some available traditional MCGDM methods. Ji et al.^36^ presented an improved elimination and choice translating reality (ELECTRE) framework based on the projection model for multi-hesitant fuzzy linguistic term sets. Wang et al.^37^ put forward a vise kriterijumska optimizacija I kompromisno resenje (VIKOR) methodology based on normalized projections with fuzzy image numbers in order to address the risk assessment of the construction project. Yue^38^ built a framework for group decision making that combines a normalized projection model with the technique for order of preference by similarity to ideal solution (TOPSIS) method. However, so far, no such research about the projection model of q-ROLS has been found.

The concept of q-ROLS^30^ is new, and some of the main issues that may be addressed are listed as follows:

1. There is a lack of some relations and important operational laws viz. complement, subtraction and division operations and thus, we are unable to cope with lots of calculations while using q-ROLSs.
2. Unbalanced situations are quite prevalent, yet all existing techniques offered so far are only capable of addressing balanced circumstances.
3. The available MCGDM techniques are based on the known weights of DMs. However, in real decision making, DMs’ weights are difficult to obtain due to the urgency of the decision making and the complication of the decision environment.
4. The existing decision ranking method has counterintuitive cases. For example, when there is a linguistic term of one criteria is equal to $\pounds _{0}$, the scoring function of the corresponding alternative will result in zero. Thus, it leads to biased results (see the details in “Heronian operators based method” section).

These challenges have prompted us to consider the following key aims for this paper.

1. To devise additional operational laws and relations to tackle the enlisted challenge 1.
2. To propose cosine and weighted cosine similarity measures in terms of linguistic scale function to cover the limitation 2.
3. To build up an MCGDM method for managing q-rung orthopair linguistic information, which can derive a ranking order with unknown weight information of DMs to resolve defects 3 and 4.

To demonstrate the applicability of the presented approach in this paper, we solve an MCGDM problem regarding EVCS site selection. In addition, we embark on a comparison to further illustrate the superiority of the new approach over the current ones.

Our research differs from previous research^25,30^ in the following ways:

1. The criteria and the DMs weight information in the existing studies are supplied in advance; however, in our study, these weights information are derived using the proposed measures of q-ROLNs.
2. No distance or similarity measures for q-ROLNs have been devised in the existing research work. Both of these goals are met in our research, and their characteristics are highlighted. As a result, our research may contribute to the theoretical foundation of q-ROLNs and encourage their broad range of applications in diverse domains.
3. The aggregation operators in the existing studies are incapable of solving MCGDM problems due to counterintuitive cases. Our study’s proposed q-rung orthopair linguistic method is the compensatory MCGDM method and reports no counterintuitive cases.

The rest of this article is arranged in the following sections. “Mathematical preliminaries” section reviews some basic concepts that are required for understanding our proposal. In “Additional operational laws and relations of q-ROLNs” section, some relations and operations of q-ROLSs are inserted. “Some similarity measures between q-rung orthopair linguistic sets” section defines q-rung orthopair linguistic cosine similarity measures and their weighted forms with a brief study of related properties. “Multi-criteria group decision making based on projection method” section offers an MCGDM method and a case study on EVCS site selection. In addition, sensitivity analysis is also performed in this section. “Comparative study” section summarizes the advantages and validity of the presented method by making a comparison with other methods. The last section draws some conclusions and point out the future research lines.

## Mathematical preliminaries

This section is devoted to recall some primary concepts about linguistic term set, q-ROLS and linguistic scale function beneficial for entire discussions.

### **Definition 1**

Herrera and Martínez^39^ Let $\pounds =\left\{ \pounds _{\alpha } | \alpha =0,1,\ldots ,\tau \right\}$ with odd cardinality be a linguistic term set (LTS), where $\pounds _{\alpha }$ have the characteristics such as

1. $$\pounds _{\alpha _1}\le \pounds _{\alpha _2} \Leftrightarrow \alpha _{1}\le \alpha _{2};$$
2. Negation$$\left( \pounds _{\alpha } \right) =\pounds _{\tau -\alpha };$$
3. $$\max \left\{ \pounds _{\alpha _1},\pounds _{\alpha _2} \right\} =\pounds _{\max \left( \alpha _1,\alpha _2 \right) };$$
4. $$\min \left\{ \pounds _{\alpha _1},\pounds _{\alpha _2} \right\} =\pounds _{\min \left( \alpha _1,\alpha _2 \right) }.$$

Later on Xu^40^ expanded this set to continuous version and defined LTS as $\overline{\pounds }=\left\{ \pounds _{\alpha } | \pounds _{0}\le \pounds _{\alpha }\le \pounds _{\tau }, \alpha \in \left[ 0,\tau \right] \right\} .$

### **Definition 2**

Yager^41^ A q-rung orthopair fuzzy set $\mathcal {A}$ on the universal set $\mathcal {Z}$ is characterized as

1 $$\begin{aligned} \mathcal{A}= \left\{ \left\langle z,\sigma \left( z \right) ,\varrho \left( z \right) \right\rangle | z \in \mathcal {Z}\right\} , \end{aligned}$$

where $\sigma ,\varrho : \mathcal {Z}\longrightarrow \left[ 0,1 \right]$ define the membership grade and non-membership grade with $\sigma ^q\left( z \right) + \varrho ^q\left( z \right) \le 1$ for $q\ge 1$ integers $\forall z \in \mathcal {Z}$. A pair $\left\langle \sigma , \varrho \right\rangle$ is called a q-ROFN.

### **Definition 3**

Yager^41^ For a q-ROFN $\mathcal {A}=\left\langle \sigma , \varrho \right\rangle$, the score function is

2 $$\begin{aligned} S\left( \mathcal {A}\right) =\sigma ^q-\varrho ^q, \end{aligned}$$

and accuracy function is

3 $$\begin{aligned} A\left( \mathcal {A}\right) =\sigma ^q+\varrho ^q, \end{aligned}$$

where $q\ge 1$ is integer.

### **Definition 4**

Yager^41^ For two q-ROFNs $\mathcal {A}_{1}$ and $\mathcal {A}_{2}$, $\mathcal {A}_{1}\succ \mathcal {A}_{2}$ where $\succ$ refers preferred to if either $S\left( \mathcal {A}_{1} \right) >S\left( \mathcal {A}_{1} \right)$ or $S\left( \mathcal {A}_{1} \right) =S\left( \mathcal {A}_{1} \right)$ and $A\left( \mathcal {A}_{1} \right) >A\left( \mathcal {A}_{1} \right)$ holds.

### **Definition 5**

Wang et al.^22^ For two q-ROFSs $\mathcal {A}_1$ and $\mathcal {A}_2$ on the universal set $\mathcal {Z}$, the q-rung orthopair fuzzy cosine similarity measure between $\mathcal {A}_1$ and $\mathcal {A}_2$ is given as

4 $$\begin{aligned} \mathcal {C}\left( \mathcal {A}_1,\mathcal {A}_2 \right) =\frac{1}{n}\sum _{j=1}^{n}\frac{\sigma ^q_1\left( z_j\right) \sigma ^q_2\left( z_j\right) + \varrho ^q_1\left( z_j\right) \varrho ^q_2\left( z_j\right) }{\sqrt{\left( \sigma ^q_1\left( z_j\right) \right) ^2+ \left( \varrho ^q_1\left( z_j\right) \right) ^2}+ \sqrt{\left( \sigma ^q_2\left( z_j\right) \right) ^2+ \left( \varrho ^q_2\left( z_j\right) \right) ^2}}. \end{aligned}$$

The cosine similarity measure $\mathcal {C}\left( \mathcal {A}_1,\mathcal {A}_2 \right)$ satisfies the following properties.

$$(p_1)$$.
$$0 \le \mathcal {C}\left( \mathcal {A}_1,\mathcal {A}_2 \right) \le 1;$$
$$(p_2)$$.
$$\mathcal {C}\left( \mathcal {A}_1,\mathcal {A}_2 \right) =\mathcal {C}\left( \mathcal {A}_2,\mathcal {A}_1 \right) ;$$
$$(p_3)$$.
$\mathcal {C}\left( \mathcal {A}_1,\mathcal {A}_2 \right) =1$ if $\mathcal {A}_1=\mathcal {A}_2$, that is, $\sigma _1(z_j)=\sigma _2(z_j)$ and $\varrho _1(z_j)=\varrho _2(z_j),$ $\forall \; j=1,2,\ldots ,n.$

It is easy to know the regular similarity measure between fuzzy sets fulfills the following Lemma 1.

### **Lemma 1**

*Let* $\mathscr {F}_1$ *and* $\mathscr {F}_2$ *be two fuzzy sets, if the similarity measure* $\mathcal {C}\left( \mathscr {F}_1, \mathscr {F}_2\right)$ *fulfills the below properties*:

$$(p_1)$$.
$$0\le \mathcal {C}\left( \mathscr {F}_1, \mathscr {F}_2\right) \le 1;$$
$$(p_2)$$.
$$\mathcal {C}\left( \mathscr {F}_1, \mathscr {F}_2\right) =\mathcal {C}\left( \mathscr {F}_2, \mathscr {F}_1\right) ;$$
$$(p_3)$$.
$\mathcal {C}\left( \mathscr {F}_1, \mathscr {F}_2\right) =1$ if and only if $\mathscr {F}_1=\mathscr {F}_2.$

### **Definition 6**

Li et al.^30^ Let $\mathcal {Z}$ be a universal set and $\overline{\pounds }$ be a continuous linguistic term set of $\pounds =\left\{ \pounds _{\alpha } | \alpha =1,2,\ldots ,\tau \right\}$, then a q-rung orthopair linguistic set (q-ROLS) $\mathcal {Q}$ is describe as

5 $$\begin{aligned} \mathcal {Q}=\left\{ \left\langle z, \pounds _{\alpha (z)},\left( \sigma (z),\varrho (z) \right) \right\rangle | z \in \mathcal {Z} \right\} , \end{aligned}$$

where $\pounds _{\alpha (z)} \in \overline{\pounds };$ $\sigma ,\varrho : \mathcal {Z}\longrightarrow \left[ 0,1 \right]$ define the membership grade and non-membership grade with $\sigma ^q\left( z \right) + \varrho ^q\left( z \right) \le 1$ for $q\ge 1$ integers $\forall z \in \mathcal {Z}$. A pair $\left\langle \sigma , \varrho \right\rangle$ is called a q-rung orthopair linguistic number (q-ROLN), which can be simply marked by $\mathcal {Q}=\left\langle \pounds _{\alpha },\left( \sigma ,\varrho \right) \right\rangle .$

### **Definition 7**

Li et al.^30^ For a q-ROLN $\mathcal {Q}=\left\langle \pounds _{\alpha },\left( \sigma ,\varrho \right) \right\rangle$, the score function is

6 $$\begin{aligned} S\left( \mathcal {Q}\right) =\left( \sigma ^q+1-\varrho ^q\right) \times \alpha , \end{aligned}$$

and accuracy function is

7 $$\begin{aligned} A\left( \mathcal {Q}\right) =\left( \sigma ^q+\varrho ^q\right) \times \alpha , \end{aligned}$$

where $q\ge 1$ is integer.

### **Definition 8**

Li et al.^30^ For two q-ROLNs $\mathcal {Q}_{1}$ and $\mathcal {Q}_{2}$, $\mathcal {Q}_{1}\succ \mathcal {Q}_{2}$ where $\succ$ refers preferred to if either $S\left( \mathcal {Q}_{1} \right) >S\left( \mathcal {Q}_{1} \right)$ or $S\left( \mathcal {Q}_{1} \right) =S\left( \mathcal {Q}_{1} \right)$ and $A\left( \mathcal {Q}_{1} \right) >A\left( \mathcal {Q}_{1} \right)$ holds.

### **Definition 9**

Li et al.^30^ For two q-ROLNs $\mathcal {Q}_{1}=\left\langle \pounds _{\alpha _1},\left( \sigma _1,\varrho _1 \right) \right\rangle$, $\mathcal {Q}_{2}= \left\langle \pounds _{\alpha _2},\left( \sigma _2,\varrho _2 \right) \right\rangle$ and positive real number $\lambda$, the basic operational laws are stated as

1. $$\mathcal {Q}_{1}\oplus \mathcal {Q}_{2}=\left\langle \pounds _{\alpha _1+\alpha _2}, \left( \left( \sigma ^{q}_{1}+\sigma ^{q}_{2}-\sigma ^{q}_{1}\sigma _{2}^{q} \right) ^{1/q},\varrho _{1}\varrho _{2}\right) \right\rangle ;$$
2. $$\mathcal {Q}_{1}\otimes \mathcal {Q}_{2}=\left\langle \pounds _{\alpha _1\times \alpha _2},\left( \sigma _{1}\sigma _{2},\left( \varrho _{1}^{q}+\varrho _{2}^{q}-\varrho _{1}^{q}\varrho _{2}^{q} \right) ^{1/q}\right) \right\rangle ;$$
3. $$\lambda \mathcal {Q}_{1}=\left\langle \pounds _{\lambda \times \alpha _1}, \left( \left( 1-\left( 1-\sigma ^q_{1}\right) ^{\lambda }\right) ^{1/q},\varrho ^{\lambda }_{1}\right) \right\rangle ;$$
4. $$\mathcal {Q}^{\lambda }_{1}=\left\langle \pounds _{\alpha ^{\lambda }_1}, \left( \sigma ^{\lambda }_{1},\left( 1-\left( 1-\varrho ^q_{1}\right) ^{\lambda }\right) ^{1/q}\right) \right\rangle .$$

### **Definition 10**

^42^ Let $\pounds =\left\{ \pounds _{\alpha } | \alpha =0,1,\ldots ,2 \tau \right\}$ be a linguistic term set. If $\vartheta _{\alpha }$ is a numeric value between 0 and 1, then the linguistic scale function *g* can be defined as follows:

8 $$\begin{aligned} g:\pounds _{\alpha }\longrightarrow \vartheta _{\alpha };\;\;\;\; g^{-1}:\vartheta _{\alpha }\longrightarrow \pounds _{\alpha } \left( \alpha =0,1,\ldots ,2 \tau \right) , \end{aligned}$$

where $0\le \vartheta _{0}< \vartheta _{1}<\cdots <\vartheta _{2 \tau }\le 1.$ The linguistic scale function is strictly monotonously increasing function with respect to the subscript of $\pounds _{\alpha }$; in fact, the function value $\vartheta _{\alpha }$ represents the semantics of the linguistic terms.

Now we discuss three commonly used linguistic scale functions as follows:

9 $$\begin{aligned} g_1\left( \pounds _{\alpha } \right) =\frac{\alpha }{2 \tau }\; \left( \alpha =0,1,\ldots 2 \tau \right) . \end{aligned}$$

The evaluation scale of the linguistic information expressed by $g_1\left( \pounds _{\alpha } \right)$ is divided on average.

10 $$\begin{aligned} g_2\left( \pounds _{\alpha } \right) ={\left\{ \begin{array}{ll} \frac{c^{\tau }-c^{\tau -\alpha }}{2c^{\tau }-2}, &{} \alpha =0,1,\ldots ,\tau ; \\ \frac{c^{\tau }+c^{\alpha -\tau }-2}{2c^{\tau }-2}, &{} \alpha =\tau +1,\tau +2,\ldots ,2\tau . \end{array}\right. } \end{aligned}$$

For linguistic scale function $g_2\left( \pounds _{\alpha } \right)$, the absolute deviation between adjacent language sets will increase when we extend it from the middle of the given set of language to both ends. Many experimental studies demonstrated that $c = 1.4$^43^.

11 $$\begin{aligned} g_3\left( \pounds _{\alpha } \right) ={\left\{ \begin{array}{ll} \frac{\tau ^{\beta _1}-\left( \tau -\alpha \right) ^{\beta _1}}{2 \tau ^{\beta _1}}, &{} \alpha =0,1,\ldots ,\tau ; \\ \frac{\tau ^{\beta _2}+\left( \alpha -\tau \right) ^{\beta _2}}{2 \tau ^{\beta _2}}, &{} \alpha =\tau +1,\tau +2,\ldots ,2\tau , \end{array}\right. } \end{aligned}$$

where $\beta _1, \beta _2 \in (0,1],$ here we assume that $\beta _1=\beta _2=0.8$^44^.

For linguistic scale function $g_3\left( \pounds _{\alpha } \right)$, the absolute deviation between adjacent language sets will decrease when we extend it from the middle of the given linguistic term set to both ends.

The above linguistic scale function can be extended to continuous linguistic scale function $g^{*}: \bar{\pounds }\longrightarrow R^+$ (where $R^+$ is a nonnegative real number between 0 and 1), which is also a strict monotonically increasing function.

### *Example 1*

Let $\bar{\pounds }=\left\{ \pounds _{\alpha } |\alpha \in \left[ 0,6 \right] \right\}$ be a continuous LTS, we can obtain the inverse of the following linguistic scale function.

If $$g^{*}\left( \pounds _{\alpha } \right) =\vartheta _{\alpha }=\frac{\alpha }{6}\left( \alpha =0,1,2,\ldots ,6\right) ,$$ then $${g^{*}}^{-1}\left( \vartheta _{\alpha }\right) =\pounds _{6 \vartheta _{\alpha }}\left( \vartheta _{\alpha } \in \left[ 0,1 \right] \right) .$$

$$\text {If } g^{*}\left( \pounds _{\alpha } \right) =\vartheta _{\alpha }={\left\{ \begin{array}{ll} \frac{c^{\tau }-c^{\tau -\alpha }}{2c^{\tau }-2}, &{} \alpha =0,1,2,3; \\ \frac{c^{\tau }+c^{\alpha -\tau }-2}{2c^{\tau }-2}, &{} \alpha =4,5,6, \end{array}\right. }$$ then $${g^{*}}^{-1}\left( \vartheta _{\alpha }\right) ={\left\{ \begin{array}{ll} \pounds _{3-\log _c\left[ c^3-\left( 2c^3-2 \right) \vartheta _{\alpha } \right] }, &{} \vartheta _{\alpha }\in \left[ 0,\frac{1}{2} \right] ; \\ \pounds _{3+\log _c\left[ \left( 2c^3-2 \right) \vartheta _{\alpha } -c^3+2\right] }, &{} \vartheta _{\alpha }\in \left( \frac{1}{2} ,1\right] . \end{array}\right. }$$

$$\text {If } g^{*}\left( \pounds _{\alpha } \right) =\vartheta _{\alpha }={\left\{ \begin{array}{ll} \frac{3^{\beta _1}-\left( 3-\alpha \right) ^{\beta _1}}{2 \times 3^{\beta _1}}, &{} \alpha =0,1,2,3; \\ \frac{3^{\beta _2}+\left( \alpha -3\right) ^{\beta _2}}{2\times 3^{\beta _2}}, &{} \alpha =4,5,6, \end{array}\right. }$$ then $${g^{*}}^{-1}\left( \vartheta _{\alpha }\right) ={\left\{ \begin{array}{ll} \pounds _{3-\left[ 3^{\beta _1}-2 \times 3^{\beta _1}\vartheta _{\alpha }\right] ^{1/\beta _1}}, &{} \vartheta _{\alpha }\in \left[ 0,1 \right] , \\ \pounds _{3+\left[ 2 \times 3^{\beta _2}\vartheta _{\alpha }-3^{\beta _2}\right] ^{1/\beta _2}}, &{} \vartheta _{\alpha }\in \left( \frac{1}{2},1 \right] . \end{array}\right. }$$

## Additional operational laws and relations of q-ROLNs

This section mainly focuses on additional operational laws and relations of q-ROLNs to enrich the theory.

### **Definition 11**

Let $\mathcal {Q}_{1}=\left\langle \pounds _{\alpha _1},\left( \sigma _1,\varrho _1 \right) \right\rangle$,and $\mathcal {Q}_{2}= \left\langle \pounds _{\alpha _2},\left( \sigma _2,\varrho _2 \right) \right\rangle$ be two q-ROLNs, then the following operations are defined:

1. $$\mathcal {Q}_{1}\bigcup \mathcal {Q}_{2}=\left\langle \max \left( \pounds _{\alpha _1},\pounds _{\alpha _2}\right) , \left( \max \left( \sigma _{1},\sigma _{2}\right) , \min \left( \varrho _{1},\varrho _{2}\right) \right) \right\rangle ;$$
2. $$\mathcal {Q}_{1}\bigcap \mathcal {Q}_{2}=\left\langle \min \left( \pounds _{\alpha _1},\pounds _{\alpha _2}\right) , \left( \min \left( \sigma _{1},\sigma _{2}\right) , \max \left( \varrho _{1},\varrho _{2}\right) \right) \right\rangle ;$$
3. $$\mathcal {Q}^c_{1}=\left\langle \pounds _{2\tau -\alpha _1},\left( \varrho _1,\sigma _1 \right) \right\rangle ;$$
4. $$\mathcal {Q}_{1}\subseteq \mathcal {Q}_{2}$$ if $$\alpha _1 \le \alpha _2$$, $$\sigma _{1}\le \sigma _{2}$$ and $$\varrho _1\ge \varrho _2$$;
5. $$\mathcal {Q}_{1}= \mathcal {Q}_{2}$$ if $$\alpha _1 = \alpha _2$$, $$\sigma _{1}= \sigma _{2}$$ and $$\varrho _1= \varrho _2$$.

### *Example 2*

Let $\mathcal {Q}_{1}=\left\langle \pounds _{2},\left( 0.1,0.3 \right) \right\rangle$, $\mathcal {Q}_{2}= \left\langle \pounds _{3},\left( 0.5,0.4 \right) \right\rangle$ be two q-ROLNs with $q=1$, and $\pounds =\left\{ \pounds _{0}= \text {extremely poor},\pounds _{1}=\text { very poor},\pounds _{2}=\text {poor},\pounds _{3}=\text {fair},\right. \left. \pounds _{4}=\text { good},\pounds _{5}=\text { very good}, \pounds _{6}=\text { extremely good}\right\}$ be LTS. Then

$$\mathcal {Q}_{1}\bigcup \mathcal {Q}_{2}=\left\langle \pounds _{3},\left( 0.5,0.3 \right) \right\rangle ,$$ $$\mathcal {Q}_{1}\bigcap \mathcal {Q}_{2}= \left\langle \pounds _{2},\left( 0.1,0.4 \right) \right\rangle ,$$ and $$\mathcal {Q}^c_{1}= \left\langle \pounds _{4},\left( 0.3,0.1 \right) \right\rangle .$$

### **Theorem 1**

(De Morgans’ law) *Let* $\mathcal {Q}_{1}=\left\langle \pounds _{\alpha _1},\left( \sigma _1,\varrho _1 \right) \right\rangle$, *and* $\mathcal {Q}_{2}= \left\langle \pounds _{\alpha _2},\left( \sigma _2,\varrho _2 \right) \right\rangle$ *be two q-ROLNs, then*

1. $$\left( \mathcal {Q}_{1}\bigcup \mathcal {Q}_{2}\right) ^c=\mathcal {Q}^c_{1}\bigcap \mathcal {Q}^c_{2};$$
2. $$\left( \mathcal {Q}_{1}\bigcap \mathcal {Q}_{2}\right) ^c=\mathcal {Q}^c_{1}\bigcup \mathcal {Q}^c_{2}.$$

### *Proof*

1. Since, $\mathcal {Q}_{1}\bigcup \mathcal {Q}_{2}=\left\langle \max \left( \pounds _{\alpha _1},\pounds _{\alpha _2}\right) , \left( \max \left( \sigma _{1},\sigma _{2}\right) , \min \left( \varrho _{1},\varrho _{2}\right) \right) \right\rangle .$ There are eight different possible cases, but we discuss only one of them. Taking $\max \left( \pounds _{\alpha _1},\pounds _{\alpha _2}\right) =\pounds _{\alpha _1}$, $\max \left( \sigma _{1},\sigma _{2}\right) =\sigma _{1}$, and $\min \left( \varrho _{1},\varrho _{2}\right) =\varrho _{2}$. This implies that $\mathcal {Q}_{1}\bigcup \mathcal {Q}_{2}=\left\langle \pounds _{\alpha _1} , \left( \sigma _{1}, \varrho _{2}\right) \right\rangle$. Form this, we have $\left( \mathcal {Q}_{1}\bigcup \mathcal {Q}_{2}\right) ^c=\left\langle \pounds _{2\tau -\alpha _1} , \left( \varrho _{2}, \sigma _{1}\right) \right\rangle$.
  Next, according to the complement operation $\mathcal {Q}^c_{1}=\left\langle \pounds _{2\tau -\alpha _1}, \left( \varrho _{1}, \sigma _{1} \right) \right\rangle$ and
  $\mathcal {Q}^c_{2}=\left\langle \pounds _{2\tau -\alpha _2}, \left( \varrho _{2}, \sigma _{2} \right) \right\rangle$. This implies that $\mathcal {Q}^c_{1}\bigcap \mathcal {Q}^c_{2}=\left\langle \pounds _{2\tau -\alpha _1} , \left( \varrho _{2}, \sigma _{1}\right) \right\rangle .$ Hence, we get the desired result. Analogously, we can obtain the required result for the remaining seven cases.
2. Based on the lines of part (1), we can easily get the required proof.

$$\square $$

### **Theorem 2**

*The complement operation is involutive, i.e*., $\left( \mathcal {Q}^c_{1}\right) ^c=\mathcal {Q}_{1}$.

### *Proof*

It trivially follows from the defined complement operation given in Definition 11. $\square$

The q-ROLS originally proposed by Li et al.^30^, is an efficient tool to express the uncertainty of DMs. Very limited work has been done on this context so far, particularly, no one has focused on the subtraction and division operations over q-ROLSs. These governing operations are significantly vital in exploring the integral theoretical framework of q-ROLSs. Meanwhile, these are also indispensable foundation in introducing some popular decision ranking techniques like PROMETHEE^45^ with q-rung orthopair linguistic data. Inspired by the defined operational laws of Gou and Xu^46^, in the following, we devised the subtraction and division operational laws for q-ROLNs.

### **Definition 12**

For any two $\mathcal {Q}_{1}=\left\langle \pounds _{\alpha _1},\left( \sigma _1,\varrho _1 \right) \right\rangle$,and $\mathcal {Q}_{2}= \left\langle \pounds _{\alpha _2},\left( \sigma _2,\varrho _2 \right) \right\rangle$ q-ROLNs, the substraction and division operations have the following forms:

1. $$\mathcal {Q}_{1}\ominus \mathcal {Q}_{2}=\left\langle g^{-1}\left( \frac{g\left( \pounds _{\alpha _1}\right) - g\left( \pounds _{\alpha _2}\right) }{1-g\left( \pounds _{\alpha _2}\right) } \right) , \left( \root q \of {\frac{\sigma ^q_{1}-\sigma ^q_{2}}{1-\sigma ^q_{2}}}, \frac{\varrho _{1}}{\varrho _{2}} \right) \right\rangle ;$$ if $$g\left( \pounds _{\alpha _1}\right) \ge g\left( \pounds _{\alpha _2}\right) $$ and $$g\left( \pounds _{\alpha _2}\right) \ne 1$$, $$\sigma _{1}\ge \sigma ^q_{2}, \varrho _{1}\le \min \left\{ \varrho _{2}, \frac{\varrho _{2} \root q \of {1-\sigma ^q_{1}-\varrho ^q_{1}}}{\root q \of {1-\sigma ^q_{2}-\varrho ^q_{2}}}\right\} ;$$
2. $$\mathcal {Q}_{1}\oslash \mathcal {Q}_{2}=\left\langle g^{-1}\left( \frac{g\left( \pounds _{\alpha _1}\right) }{g\left( \pounds _{\alpha _2}\right) } \right) , \left( \frac{\sigma _{1}}{\sigma _{2}}, \root q \of {\frac{\varrho ^q_{1}-\varrho ^q_{2}}{1-\varrho ^q_{2}}} \right) \right\rangle ;$$ if $$g\left( \pounds _{\alpha _1}\right) \le g\left( \pounds _{\alpha _2}\right) $$ and $$g\left( \pounds _{\alpha _2}\right) \ne 0$$, $$\varrho _{1}\ge \varrho ^q_{2}, \sigma _{1}\le \min \left\{ \sigma _{2}, \frac{\sigma _{2} \root q \of {1-\varrho ^q_{1}-\sigma ^q_{1}}}{\root q \of {1-\varrho ^q_{2}-\sigma ^q_{2}}}\right\} .$$

### *Example 3*

Let $\mathcal {Q}_{1}=\left\langle \pounds _{2},\left( 0.1,0.3 \right) \right\rangle$, $\mathcal {Q}_{2}= \left\langle \pounds _{3},\left( 0.5,0.4 \right) \right\rangle$, $\mathcal {Q}_{3}= \left\langle \pounds _{3},\left( 0.2,0.7 \right) \right\rangle$, $\mathcal {Q}_{4}= \left\langle \pounds _{4},\left( 0.5,0.3 \right) \right\rangle$ be q-ROLNs with $q=1$, $\pounds =\left\{ \pounds _{0}= \text {extremely poor},\pounds _{1}=\text { very poor},\pounds _{2}=\text {poor},\pounds _{3}=\text {fair}, \pounds _{4}=\text { good},\right. \left. \pounds _{5}=\text { very good}, \pounds _{6}=\text { extremely good}\right\}$ be LTS and $g^{*}=\frac{\alpha }{2 \tau }$ be linguistic scale function. Then, one can easily check that $\mathcal {Q}_{1}$, $\mathcal {Q}_{2}$ and $\mathcal {Q}_{3}$, $\mathcal {Q}_{4}$ meet the requirement of $\mathcal {Q}_{1}\ominus \mathcal {Q}_{2}$, and $\mathcal {Q}_{3}\oslash \mathcal {Q}_{4}$, respectively. Thus, $\mathcal {Q}_{1}\ominus \mathcal {Q}_{2}=\left\langle \pounds _{\frac{3}{2}},\left( 0.4444,0.75 \right) \right\rangle$ and $\mathcal {Q}_{3}\oslash \mathcal {Q}_{4}=\left\langle \pounds _{\frac{9}{2}},\left( 0.04,0.5714 \right) \right\rangle .$

## Some similarity measures between q-rung orthopair linguistic sets

This section presents the similarity measures for q-ROLs based on linguistic scale function to consider the semantics of linguistic terms. Also, we prove a couple of theorems that describe certain interesting properties of the similarity measures.

### **Definition 13**

Let $\mathcal {Q}_{1}=\left\{ \left\langle z_j, \pounds _{\alpha _1(z_j)},\left( \sigma _1(z_j),\varrho _1(z_j) \right) \right\rangle | z_j \in \mathcal {Z} \right\}$ and $\mathcal {Q}_{2}=\left\{ \left\langle z_j, \pounds _{\alpha _2(z_j)},\left( \sigma _2(z_j),\varrho _2(z_j) \right) \right\rangle | z_j \in \mathcal {Z} \right\}$ be two q-ROLSs on $\mathcal {Z}=\left\{ z_1,z_2,\ldots ,z_n \right\}$, $\pounds _{\alpha _1(z_j)}$, $\pounds _{\alpha _2(z_j)}\in \pounds =\left\{ \pounds _0,\pounds _1,\ldots ,\pounds _{2 \tau } \right\}$, and let $g^{*}$ be a linguistic scale function, then the cosine similarity measure between $\mathcal {Q}_{1}$ and $\mathcal {Q}_{2}$ is denoted and defined as

12 $$\begin{aligned} \mathcal {C}_{ql}\left( \mathcal {Q}_{1},\mathcal {Q}_{2}\right) =\frac{1}{n}\sum ^{n}_{j=1} \frac{\left( g^{*}\left( \pounds _{\alpha _1\left( z_j \right) }\right) \sigma ^q_1\left( z_j \right) g^{*}\left( \pounds _{\alpha _2\left( z_j \right) }\right) \sigma ^q_2\left( z_j \right) \right) +\left( g^{*}\left( \pounds _{\alpha _1\left( z_j \right) }\right) \varrho ^q_1\left( z_j \right) g^{*}\left( \pounds _{\alpha _2\left( z_j \right) }\right) \varrho ^q_2\left( z_j \right) \right) }{\sqrt{\left( g^{*}\left( \pounds _{\alpha _1\left( z_j\right) }\right) \sigma ^q_1\left( z_j\right) \right) ^{2}+\left( g^{*}\left( \pounds _{\alpha _1\left( z_j\right) }\right) \varrho ^q_1\left( z_j\right) \right) ^{2}} \sqrt{\left( g^{*}\left( \pounds _{\alpha _2\left( z_j\right) }\right) \sigma ^q_2\left( z_j\right) \right) ^{2}+\left( g^{*}\left( \pounds _{\alpha _2\left( z_j\right) }\right) \varrho ^q_2\left( z_j\right) \right) ^{2}}}, \end{aligned}$$

### **Theorem 3**

*The cosine similarity measure* $\mathcal {C}_{ql}\left( \mathcal {Q}_{1},\mathcal {Q}_{2}\right)$ *between* $\mathcal {Q}_{1}$ and $\mathcal {Q}_{2}$ *fulfills the following properties*:

$$(p_1)$$.
$$0 \le \mathcal {C}_{ql}\left( \mathcal {Q}_1,\mathcal {Q}_2 \right) \le 1;$$
$$(p_2)$$.
$$\mathcal {C}_{ql}\left( \mathcal {Q}_1,\mathcal {Q}_2 \right) =\mathcal {C}_{ql}\left( \mathcal {Q}_2,\mathcal {Q}_1 \right) ;$$
$$(p_3)$$.
$\mathcal {C}_{ql}\left( \mathcal {Q}_1,\mathcal {Q}_2 \right) =1$ if $\mathcal {Q}_1=\mathcal {Q}_2$, that is, $\alpha _1(z_j)=\alpha _2(z_j)$, $\sigma _1(z_j)=\sigma _2(z_j)$ and $\varrho _1(z_j)=\varrho _2(z_j),$ $\forall \; j=1,2,\ldots ,n.$
$$(p_4)$$.
$$\mathcal {C}_{ql}\left( \mathcal {Q}_1,\mathcal {Q}_3 \right) \le \mathcal {C}_{ql}\left( \mathcal {Q}_1,\mathcal {Q}_2 \right) +\mathcal {C}_{ql}\left( \mathcal {Q}_2,\mathcal {Q}_3 \right) $$ if $$\mathcal {Q}_1\subseteq \mathcal {Q}_2\subseteq \mathcal {Q}_3.$$

### *Proof*

$$(p_1)$$.
According to Eq. (12) it is clear that $\mathcal {C}_{ql}\left( \mathcal {Q}_1,\mathcal {Q}_2 \right) \ge 0.$ Further, $\frac{1}{n}\sum ^{n}_{j=1} \frac{\left( g^{*}\left( \pounds _{\alpha _1\left( z_j \right) }\right) \sigma ^q_1\left( z_j \right) g^{*}\left( \pounds _{\alpha _2\left( z_j \right) }\right) \sigma ^q_2\left( z_j \right) \right) +\left( g^{*}\left( \pounds _{\alpha _1\left( z_j \right) }\right) \varrho ^q_1\left( z_j \right) g^{*}\left( \pounds _{\alpha _2\left( z_j \right) }\right) \varrho ^q_2\left( z_j \right) \right) }{\sqrt{\left( g^{*}\left( \pounds _{\alpha _1\left( z_j\right) }\right) \sigma ^q_1\left( z_j\right) \right) ^{2}+\left( g^{*}\left( \pounds _{\alpha _1\left( z_j\right) }\right) \varrho ^q_1\left( z_j\right) \right) ^{2}} \sqrt{\left( g^{*}\left( \pounds _{\alpha _2\left( z_j\right) }\right) \sigma ^q_2\left( z_j\right) \right) ^{2}+\left( g^{*}\left( \pounds _{\alpha _2\left( z_j\right) }\right) \varrho ^q_2\left( z_j\right) \right) ^{2}}} \le 1$, according to the range of cosine function value. Thus, $0\le \mathcal {C}_{ql}\left( \mathcal {Q}_1,\mathcal {Q}_2 \right) \le 1$.
$$(p_2)$$.
Since, $$\mathcal {C}_{ql}\left( \mathcal {Q}_{1},\mathcal {Q}_{2}\right) = \frac{1}{n}\sum ^{n}_{j=1} \frac{\left( g^{*}\left( \pounds _{\alpha _1\left( z_j \right) }\right) \sigma ^q_1\left( z_j \right) g^{*}\left( \pounds _{\alpha _2\left( z_j \right) }\right) \sigma ^q_2\left( z_j \right) \right) +\left( g^{*}\left( \pounds _{\alpha _1\left( z_j \right) }\right) \varrho ^q_1\left( z_j \right) g^{*}\left( \pounds _{\alpha _2\left( z_j \right) }\right) \varrho ^q_2\left( z_j \right) \right) }{\sqrt{\left( g^{*}\left( \pounds _{\alpha _1\left( z_j\right) }\right) \sigma ^q_1\left( z_j\right) \right) ^{2}+\left( g^{*}\left( \pounds _{\alpha _1\left( z_j\right) }\right) \varrho ^q_1\left( z_j\right) \right) ^{2}} \sqrt{\left( g^{*}\left( \pounds _{\alpha _2\left( z_j\right) }\right) \sigma ^q_2\left( z_j\right) \right) ^{2}+\left( g^{*}\left( \pounds _{\alpha _2\left( z_j\right) }\right) \varrho ^q_2\left( z_j\right) \right) ^{2}}}=$$ $$\frac{1}{n}\sum ^{n}_{j=1} \frac{\left( g^{*}\left( \pounds _{\alpha _2\left( z_j \right) }\right) \sigma ^q_2\left( z_j \right) g^{*}\left( \pounds _{\alpha _1\left( z_j \right) }\right) \sigma ^q_1\left( z_j \right) \right) +\left( g^{*}\left( \pounds _{\alpha _2\left( z_j \right) }\right) \varrho ^q_2\left( z_j \right) g^{*}\left( \pounds _{\alpha _1\left( z_j \right) }\right) \varrho ^q_1\left( z_j \right) \right) }{\sqrt{\left( g^{*}\left( \pounds _{\alpha _1\left( z_j\right) }\right) \sigma ^q_2\left( z_j\right) \right) ^{2}+\left( g^{*}\left( \pounds _{\alpha _2\left( z_j\right) }\right) \varrho ^q_2\left( z_j\right) \right) ^{2}} \sqrt{\left( g^{*}\left( \pounds _{\alpha _1\left( z_j\right) }\right) \sigma ^q_1\left( z_j\right) \right) ^{2}+\left( g^{*}\left( \pounds _{\alpha _1\left( z_j\right) }\right) \varrho ^q_1\left( z_j\right) \right) ^{2}}}= \mathcal {C}_{ql}\left( \mathcal {Q}_2,\mathcal {Q}_1 \right) .$$
$$(p_3)$$.
If $\mathcal {Q}_1=\mathcal {Q}_2$, that is, $\alpha _1(z_j)=\alpha _2(z_j)$, $\sigma _1(z_j)=\sigma _2(z_j)$ and $\varrho _1(z_j)=\varrho _2(z_j),$ then $\mathcal {C}_{ql}\left( \mathcal {Q}_{1},\mathcal {Q}_{2}\right) =\frac{1}{n}\sum ^{n}_{j=1} \frac{\left( g^{*}\left( \pounds _{\alpha _1\left( z_j \right) }\right) \sigma ^q_1\left( z_j \right) g^{*}\left( \pounds _{\alpha _1\left( z_j \right) }\right) \sigma ^q_1\left( z_j \right) \right) +\left( g^{*}\left( \pounds _{\alpha _1\left( z_j \right) }\right) \varrho ^q_1\left( z_j \right) g^{*}\left( \pounds _{\alpha _1\left( z_j \right) }\right) \varrho ^q_1\left( z_j \right) \right) }{\sqrt{\left( g^{*}\left( \pounds _{\alpha _1\left( z_j\right) }\right) \sigma ^q_1\left( z_j\right) \right) ^{2}+\left( g^{*}\left( \pounds _{\alpha _1\left( z_j\right) }\right) \varrho ^q_1\left( z_j\right) \right) ^{2}} \sqrt{\left( g^{*}\left( \pounds _{\alpha _1\left( z_j\right) }\right) \sigma ^q_1\left( z_j\right) \right) ^{2}+\left( g^{*}\left( \pounds _{\alpha _1\left( z_j\right) }\right) \varrho ^q_1\left( z_j\right) \right) ^{2}}}=1.$
$$(p_4)$$.
Since $$\mathcal {C}_{ql}\left( \mathcal {Q}_{1}\left( z_j\right) ,\mathcal {Q}_{2}\left( z_j\right) \right) =$$
  $$\begin{aligned} \frac{\left( g^{*}\left( \pounds _{\alpha _1\left( z_j \right) }\right) \sigma ^q_1\left( z_j \right) g^{*}\left( \pounds _{\alpha _2\left( z_j \right) }\right) \sigma ^q_2\left( z_j \right) \right) +\left( g^{*}\left( \pounds _{\alpha _1\left( z_j \right) }\right) \varrho ^q_1\left( z_j \right) g^{*}\left( \pounds _{\alpha _2\left( z_j \right) }\right) \varrho ^q_2\left( z_j \right) \right) }{\sqrt{\left( g^{*}\left( \pounds _{\alpha _1\left( z_j\right) }\right) \sigma ^q_1\left( z_j\right) \right) ^{2}+\left( g^{*}\left( \pounds _{\alpha _1\left( z_j\right) }\right) \varrho ^q_1\left( z_j\right) \right) ^{2}} \sqrt{\left( g^{*}\left( \pounds _{\alpha _2\left( z_j\right) }\right) \sigma ^q_2\left( z_j\right) \right) ^{2}+\left( g^{*}\left( \pounds _{\alpha _2\left( z_j\right) }\right) \varrho ^q_2\left( z_j\right) \right) ^{2}}}, \end{aligned}$$
  $$\mathcal {C}_{ql}\left( \mathcal {Q}_{2}\left( z_j\right) ,\mathcal {Q}_{3}\left( z_j\right) \right) =$$
  $$\begin{aligned} \frac{\left( g^{*}\left( \pounds _{\alpha _2\left( z_j \right) }\right) \sigma ^q_2\left( z_j \right) g^{*}\left( \pounds _{\alpha _3\left( z_j \right) }\right) \sigma ^q_3\left( z_j \right) \right) +\left( g^{*}\left( \pounds _{\alpha _2\left( z_j \right) }\right) \varrho ^q_2\left( z_j \right) g^{*}\left( \pounds _{\alpha _3\left( z_j \right) }\right) \varrho ^q_3\left( z_j \right) \right) }{\sqrt{\left( g^{*}\left( \pounds _{\alpha _2\left( z_j\right) }\right) \sigma ^q_2\left( z_j\right) \right) ^{2}+\left( g^{*}\left( \pounds _{\alpha _2\left( z_j\right) }\right) \varrho ^q_1\left( z_j\right) \right) ^{2}} \sqrt{\left( g^{*}\left( \pounds _{\alpha _3\left( z_j\right) }\right) \sigma ^q_3\left( z_j\right) \right) ^{2}+\left( g^{*}\left( \pounds _{\alpha _3\left( z_j\right) }\right) \varrho ^q_3\left( z_j\right) \right) ^{2}}}, \end{aligned}$$
  $$\mathcal {C}_{ql}\left( \mathcal {Q}_{1}\left( z_j\right) ,\mathcal {Q}_{3}\left( z_j\right) \right) =$$
  $$\begin{aligned} \frac{\left( g^{*}\left( \pounds _{\alpha _1\left( z_j \right) }\right) \sigma ^q_1\left( z_j \right) g^{*}\left( \pounds _{\alpha _3\left( z_j \right) }\right) \sigma ^q_3\left( z_j \right) \right) +\left( g^{*}\left( \pounds _{\alpha _1\left( z_j \right) }\right) \varrho ^q_1\left( z_j \right) g^{*}\left( \pounds _{\alpha _3\left( z_j \right) }\right) \varrho ^q_3\left( z_j \right) \right) }{\sqrt{\left( g^{*}\left( \pounds _{\alpha _1\left( z_j\right) }\right) \sigma ^q_1\left( z_j\right) \right) ^{2}+\left( g^{*}\left( \pounds _{\alpha _1\left( z_j\right) }\right) \varrho ^q_1\left( z_j\right) \right) ^{2}} \sqrt{\left( g^{*}\left( \pounds _{\alpha _3\left( z_j\right) }\right) \sigma ^q_3\left( z_j\right) \right) ^{2}+\left( g^{*}\left( \pounds _{\alpha _3\left( z_j\right) }\right) \varrho ^q_3\left( z_j\right) \right) ^{2}}}, \end{aligned}$$
  where $\mathcal {Q}_{1}\left( z_j\right)$, $\mathcal {Q}_{2}\left( z_j\right)$ and $\mathcal {Q}_{3}\left( z_j\right)$ are three vectors in one plane, if $\mathcal {Q}_{1}\left( z_j\right) \subseteq \mathcal {Q}_{1}\left( z_j\right) \subseteq \mathcal {Q}_{1}\left( z_j\right) \forall j=1,2,\ldots n$, then it is clear that $\mathcal {C}_{ql}\left( \mathcal {Q}_{1}\left( z_j\right) ,\mathcal {Q}_{3}\left( z_j\right) \right) \le$ $\mathcal {C}_{ql}\left( \mathcal {Q}_{1}\left( z_j\right) ,\mathcal {Q}_{2}\left( z_j\right) \right) +$ $\mathcal {C}_{ql}\left( \mathcal {Q}_{1}\left( z_j\right) ,\mathcal {Q}_{3}\left( z_j\right) \right)$ based on the triangle inequality. Combining the inequalities $\forall j=1,2,\ldots ,n$, we can get $\mathcal {C}_{ql}\left( \mathcal {Q}_1,\mathcal {Q}_3 \right) \le \mathcal {C}_{ql}\left( \mathcal {Q}_1,\mathcal {Q}_2 \right) +\mathcal {C}_{ql}\left( \mathcal {Q}_2,\mathcal {Q}_3 \right) .$

$$\square $$

If we consider the weight of different elements $z_j \in \mathcal {Z}$, then we introduce q-rung orthhopair linguistic weighted cosine similarity measure, which can be defined as follows:

### **Definition 14**

Let $\mathcal {Q}_{1}=\left\{ \left\langle z_j, \pounds _{\alpha _1(z_j)},\left( \sigma _1(z_j),\varrho _1(z_j) \right) \right\rangle | z_j \in \mathcal {Z} \right\}$ and $\mathcal {Q}_{2}=\left\{ \left\langle z_j, \pounds _{\alpha _2(z_j)},\left( \sigma _2(z_j),\varrho _2(z_j) \right) \right\rangle | z_j \in \mathcal {Z} \right\}$ be two q-ROLSs on $\mathcal {Z}=\left\{ z_1,z_2,\ldots ,z_n \right\}$, $\pounds _{\alpha _1(z_j)}$, $\pounds _{\alpha _2(z_j)}\in \pounds =\left\{ \pounds _0,\pounds _1,\ldots ,\pounds _{2 \tau } \right\}$, and let $g^{*}$ be a linguistic scale function. Further, if the weight of $z_i$ is $\varpi _j\left( \sum _{j=1}^{n}\varpi _j=1 \text { and } 0\le \varpi _j\le 1\right)$, then the weighted cosine similarity measure between $\mathcal {Q}_{1}$ and $\mathcal {Q}_{2}$ is denoted and defined as

13 $$\begin{aligned} \mathcal {C}_{qlw}\left( \mathcal {Q}_{1},\mathcal {Q}_{2}\right) =\sum ^{n}_{j=1}\varpi _j \frac{\left( g^{*}\left( \pounds _{\alpha _1\left( z_j \right) }\right) \sigma ^q_1\left( z_j \right) g^{*}\left( \pounds _{\alpha _2\left( z_j \right) }\right) \sigma ^q_2\left( z_j \right) \right) +\left( g^{*}\left( \pounds _{\alpha _1\left( z_j \right) }\right) \varrho ^q_1\left( z_j \right) g^{*}\left( \pounds _{\alpha _2\left( z_j \right) }\right) \varrho ^q_2\left( z_j \right) \right) }{\sqrt{\left( g^{*}\left( \pounds _{\alpha _1\left( z_j\right) }\right) \sigma ^q_1\left( z_j\right) \right) ^{2}+\left( g^{*}\left( \pounds _{\alpha _1\left( z_j\right) }\right) \varrho ^q_1\left( z_j\right) \right) ^{2}} \sqrt{\left( g^{*}\left( \pounds _{\alpha _2\left( z_j\right) }\right) \sigma ^q_2\left( z_j\right) \right) ^{2}+\left( g^{*}\left( \pounds _{\alpha _2\left( z_j\right) }\right) \varrho ^q_2\left( z_j\right) \right) ^{2}}}. \end{aligned}$$

### *Remark 1*

If the weight $\varpi _j=\frac{1}{n}$ for all j, then the weighted cosine similarity measure $\mathcal {C}_{qlw}\left( \mathcal {Q}_{1},\mathcal {Q}_{2}\right)$ is reduced to the cosine similarity measure $\mathcal {C}_{ql}\left( \mathcal {Q}_{1},\mathcal {Q}_{2}\right)$.

### **Theorem 4**

*Let* $\mathcal {Q}_{1}$, $\mathcal {Q}_{2}$ *and* $\mathcal {Q}_{3}$ *be three q-ROLSs, then the weighted cosine similarity measure* $\mathcal {C}_{qlw}\left( \mathcal {Q}_{i},\mathcal {Q}_{j}\right) (i,j=1,2,3)$ *fulfills the following properties*:

$$(p_1)$$.
$$0 \le \mathcal {C}_{qlw}\left( \mathcal {Q}_1,\mathcal {Q}_2 \right) \le 1;$$
$$(p_2)$$.
$$\mathcal {C}_{qlw}\left( \mathcal {Q}_1,\mathcal {Q}_2 \right) =\mathcal {C}_{qlw}\left( \mathcal {Q}_2,\mathcal {Q}_1 \right) ;$$
$$(p_3)$$.
$\mathcal {C}_{qlw}\left( \mathcal {Q}_1,\mathcal {Q}_2 \right) =1$ if $\mathcal {Q}_1=\mathcal {Q}_2$, that is, $\alpha _1(z_j)=\alpha _2(z_j)$, $\sigma _1(z_j)=\sigma _2(z_j)$ and $\varrho _1(z_j)=\varrho _2(z_j),$ $\forall \; j=1,2,\ldots ,n;$
$$(p_4)$$.
$$\mathcal {C}_{qlw}\left( \mathcal {Q}_1,\mathcal {Q}_3 \right) \le \mathcal {C}_{qlw}\left( \mathcal {Q}_1,\mathcal {Q}_2 \right) +\mathcal {C}_{qlw}\left( \mathcal {Q}_2,\mathcal {Q}_3 \right) $$ if $$\mathcal {Q}_1\subseteq \mathcal {Q}_2\subseteq \mathcal {Q}_3.$$

### *Proof*

Based on the lines of Theorem 3, one can easily prove these properties, therefore, we omit the proof here. $\square$

According to Lemma 1 the cosine similarity measures described in Definitions 13 and 14 are not the regular similarity measure since they do not meet the condition $p_3$ in Lemma 1, as shown in Example 4.

### *Example 4*

Let $\mathcal {Q}_{1}=\left\{ \left\langle z_1, \pounds _{2},\left( 0.1, 0.3\right) \right\rangle ,\left\langle z_2, \pounds _{5},\left( 0.2,0 \right) \right\rangle \right\}$ and $\mathcal {Q}_{2}=\left\{ \left\langle z_1, \pounds _{4},\left( 0.6,0 \right) \right\rangle ,\left\langle z_2, \pounds _{3},\left( 0.5,0.4 \right) \right\rangle \right\}$ be two q-ROLSs on $\mathcal {Z}=\left\{ z_1,z_2 \right\}$ and LTS $\pounds =\left\{ \pounds _{0}= \text {extremely poor},\pounds _{1}=\text { very poor},\pounds _{2}=\text {poor},\pounds _{3}=\text {fair},\right. \left. \pounds _{4}=\text { good},\pounds _{5}=\text { very good}, \pounds _{6}=\text { extremely good}\right\}$. If $q=1$, $g^{*}=\frac{\alpha }{2 \tau }$ and $\varpi =\left( \varpi _1,\varpi _2 \right) ^T=\left( 0.5,0.5\right)$. Then, we have $\mathcal {C}_{ql}\left( \mathcal {Q}_{1},\mathcal {Q}_{2}\right) =1$ and $\mathcal {C}_{qlw}\left( \mathcal {Q}_{1},\mathcal {Q}_{2}\right) =1$.

It is obvious that $\mathcal {Q}_{1}$ and $\mathcal {Q}_{2}$ are not equal, but $\mathcal {C}_{qlw}\left( \mathcal {Q}_{1},\mathcal {Q}_{2}\right) =\mathcal {C}_{qlw}\left( \mathcal {Q}_{1},\mathcal {Q}_{2}\right) =1$, which means that the cosine similarity measures $\mathcal {C}_{ql}$ and $\mathcal {C}_{qlw}$ are not regular.

If the cosine similarity measure $\mathcal {C}_{qlw}$ is employed to MCDM problems, the findings will be unfruitful. To address this issue, we need to enhance the cosine similarity measures provided in Definitions 13 and 14. We put forward a novel q-rung orthopair fuzzy linguistic similarity measure based on the presented cosine similarity measure $\mathcal {C}_{qlw}\left( \mathcal {Q}_{1},\mathcal {Q}_{2}\right)$ and the following Minkowski distance measure $\mathcal {D}_{qlw}\left( \mathcal {Q}_{1},\mathcal {Q}_{2}\right)$.

### **Definition 15**

Let $\mathcal {Q}_{1}=\left\{ \left\langle z_j, \pounds _{\alpha _1(z_j)},\left( \sigma _1(z_j),\varrho _1(z_j) \right) \right\rangle | z_j \in \mathcal {Z} \right\}$ and $\mathcal {Q}_{2}=\left\{ \left\langle z_j, \pounds _{\alpha _2(z_j)},\left( \sigma _2(z_j),\varrho _2(z_j) \right) \right\rangle | z_j \in \mathcal {Z} \right\}$ be two q-ROLSs on $\mathcal {Z}=\left\{ z_1,z_2,\ldots ,z_n \right\}$, $\pounds _{\alpha _1(z_j)}$, $\pounds _{\alpha _2(z_j)}\in \pounds =\left\{ \pounds _0,\pounds _1,\ldots ,\pounds _{2 \tau } \right\}$, and let $g^{*}$ be a linguistic scale function, then the Minkowski distance measure between $\mathcal {Q}_{1}$ and $\mathcal {Q}_{2}$ is denoted and defined as

14 $$\begin{aligned} \mathcal {D}_{ql}\left( \mathcal {Q}_{1},\mathcal {Q}_{2}\right) = \left[ \frac{1}{3n}\sum _{j=1}^{n}\left( \left| \left( g^{*}\left( \pounds _{\alpha _1\left( z_j \right) }\right) -g^{*}\left( \pounds _{\alpha _2\left( z_j \right) }\right) \right) \right| ^{\lambda } +\left| \left( \sigma ^q_1\left( z_j \right) -\sigma ^q_2\left( z_j \right) \right) \right| ^{\lambda }+\left| \left( \varrho ^q_1\left( z_j \right) -\varrho ^q_2\left( z_j \right) \right) \right| ^{\lambda }\right) \right] ^{1/\lambda }. \end{aligned}$$

If we consider the weight of different elements $z_j \in \mathcal {Z}$, then we introduce q-rung orthhopair linguistic weighted Minkowski distance measure, which can be defined as follows.

### **Definition 16**

Let $\mathcal {Q}_{1}=\left\{ \left\langle z_j, \pounds _{\alpha _1(z_j)},\left( \sigma _1(z_j),\varrho _1(z_j) \right) \right\rangle | z_j \in \mathcal {Z} \right\}$ and $\mathcal {Q}_{2}=\left\{ \left\langle z_j, \pounds _{\alpha _2(z_j)},\left( \sigma _2(z_j),\varrho _2(z_j) \right) \right\rangle | z_j \in \mathcal {Z} \right\}$ be two q-ROLSs on $\mathcal {Z}=\left\{ z_1,z_2,\ldots ,z_n \right\}$, $\pounds _{\alpha _1(z_j)}$, $\pounds _{\alpha _2(z_j)}\in \pounds =\left\{ \pounds _0,\pounds _1,\ldots ,\pounds _{2 \tau } \right\}$, and let $g^{*}$ be a linguistic scale function. Further, if the weight of $z_i$ is $\varpi _j\left( \sum _{j=1}^{n}\varpi _j=1 \text { and } 0\le \varpi _j\le 1\right)$, then weighted Minkowski distance measure between $\mathcal {Q}_{1}$ and $\mathcal {Q}_{2}$ is denoted and defined as

15 $$\begin{aligned} \mathcal {D}_{qlw}\left( \mathcal {Q}_{1},\mathcal {Q}_{2}\right) = \left[ \frac{1}{3}\sum _{j=1}^{n}\varpi _j\left( \left| \left( g^{*}\left( \pounds _{\alpha _1\left( z_j \right) }\right) -g^{*}\left( \pounds _{\alpha _2\left( z_j \right) }\right) \right) \right| ^{\lambda } +\left| \left( \sigma ^q_1\left( z_j \right) -\sigma ^q_2\left( z_j \right) \right) \right| ^{\lambda }+\left| \left( \varrho ^q_1\left( z_j \right) -\varrho ^q_2\left( z_j \right) \right) \right| ^{\lambda }\right) \right] ^{1/\lambda }. \end{aligned}$$

### *Remark 2*

If $\lambda = 1$, the distance measure $\mathcal {D}_{qlw}$ is reduced to the following Hamming distance measure $\mathcal {D}_{qlwh}$:

16 $$\begin{aligned} \mathcal {D}_{qlwh}\left( \mathcal {Q}_{1},\mathcal {Q}_{2}\right) = \frac{1}{3}\sum _{j=1}^{n}\varpi _j\left( \left| \left( g^{*}\left( \pounds _{\alpha _1\left( z_j \right) }\right) -g^{*}\left( \pounds _{\alpha _2\left( z_j \right) }\right) \right) \right| +\left| \left( \sigma ^q_1\left( z_j \right) -\sigma ^q_2\left( z_j \right) \right) \right| +\left| \left( \varrho ^q_1\left( z_j \right) -\varrho ^q_2\left( z_j \right) \right) \right| \right) . \end{aligned}$$

### *Remark 3*

If $\lambda = 2$, the distance measure $\mathcal {D}_{qlw}$ is reduced to the following Euclidean distance measure $\mathcal {D}_{qlwe}$:

17 $$\begin{aligned} \mathcal {D}_{qlwe}\left( \mathcal {Q}_{1},\mathcal {Q}_{2}\right) = \left[ \frac{1}{3}\sum _{j=1}^{n}\varpi _j\left( \left| \left( g^{*}\left( \pounds _{\alpha _1\left( z_j \right) }\right) -g^{*}\left( \pounds _{\alpha _2\left( z_j \right) }\right) \right) \right| ^{2} +\left| \left( \sigma ^q_1\left( z_j \right) -\sigma ^q_2\left( z_j \right) \right) \right| ^{2}+\left| \left( \varrho ^q_1\left( z_j \right) -\varrho ^q_2\left( z_j \right) \right) \right| ^{2}\right) \right] ^{1/2}. \end{aligned}$$

### **Theorem 5**

*Let* $\mathcal {Q}_{i}=\left\{ \left\langle z_j, \pounds _{\alpha _i(z_j)},\left( \sigma _i(z_j),\varrho _i(z_j) \right) \right\rangle | z_j \in \mathcal {Z} \right\} \left( i=1,2,3 \right)$ *be three q-ROLSs on* $\mathcal {Z}=\left\{ z_1,z_2,\ldots ,z_n \right\}$, $\pounds _{\alpha _1(z_j)}$, $\pounds _{\alpha _2(z_j)}\in \pounds =\left\{ \pounds _0,\pounds _1,\ldots ,\pounds _{2 \tau } \right\}$, and let $g^{*}$ *be a linguistic scale function. Then the weighted Minkowski distance* $\mathcal {D}_{qlw}$ *meets the following properties*:

$$(p_1)$$.
$$0 \le \mathcal {D}_{qlw}\left( \mathcal {Q}_1,\mathcal {Q}_2 \right) \le 1;$$
$$(p_2)$$.
$$\mathcal {D}_{qlw}\left( \mathcal {Q}_1,\mathcal {Q}_2 \right) =\mathcal {D}_{qlw}\left( \mathcal {Q}_2,\mathcal {Q}_1 \right) ;$$
$$(p_3)$$.
$\mathcal {D}_{qlw}\left( \mathcal {Q}_1,\mathcal {Q}_2 \right) =0$ if and only if $\mathcal {Q}_1=\mathcal {Q}_2$, that is, $\alpha _1(z_j)=\alpha _2(z_j)$, $\sigma _1(z_j)=\sigma _2(z_j)$ and $\varrho _1(z_j)=\varrho _2(z_j),$ $\forall \; j=1,2,\ldots ,n;$
$$(p_4)$$.
$$\mathcal {D}_{qlw}\left( \mathcal {Q}_1,\mathcal {Q}_3 \right) \le \mathcal {D}_{qlw}\left( \mathcal {Q}_1,\mathcal {Q}_2 \right) +\mathcal {D}_{qlw}\left( \mathcal {Q}_2,\mathcal {Q}_3 \right) $$ if $$\mathcal {Q}_1\subseteq \mathcal {Q}_2\subseteq \mathcal {Q}_3.$$

### *Proof*

$(p_1)$, $(p_2)$ and $(p_4)$ are obvious, thereby we omit their proof.

$$(p_3).$$
If $\mathcal {Q}_1=\mathcal {Q}_2$, then $g^*\left( \pounds _{\alpha _1(z_j)}\right) =g^*\left( \pounds _{\alpha _2(z_j)}\right)$, $\sigma _1(z_j)=\sigma _2(z_j)$ and $\varrho _1(z_j)=\varrho _2(z_j).$ So, $\mathcal {D}_{qlw}\left( \mathcal {Q}_1,\mathcal {Q}_2 \right) =0$ is obtained.
  Conversely, if $\mathcal {D}_{qlw}\left( \mathcal {Q}_1,\mathcal {Q}_2 \right) =0$, we have $g^{*}\left( \pounds _{\alpha _1\left( z_j \right) }\right) =g^{*}\left( \pounds _{\alpha _2\left( z_j \right) }\right)$, $\sigma ^q_1\left( z_j \right) =\sigma ^q_2\left( z_j \right)$ and $\varrho ^q_1\left( z_j \right) =\varrho ^q_2\left( z_j \right) .$ Since, the linguistic scale function $g^*$ is monotonic. Therefore, $\pounds _{\alpha _1\left( z_j \right) }=\pounds _{\alpha _2\left( z_j \right) }$ are obtained.

$$\square $$

In what follows, we define a novel similarity measure between q-ROLSs based on the linguistic scale function, which connects the proposed cosine similarity measure $\mathcal {C}_{qlw}$ and the distance measure $\mathcal {D}_{qlw}$ together.

### **Definition 17**

Let $\mathcal {Q}_{1}=\left\{ \left\langle z_j, \pounds _{\alpha _1(z_j)},\left( \sigma _1(z_j),\varrho _1(z_j) \right) \right\rangle | z_j \in \mathcal {Z} \right\}$ and $\mathcal {Q}_{2}=\left\{ \left\langle z_j, \pounds _{\alpha _2(z_j)},\left( \sigma _2(z_j),\varrho _2(z_j) \right) \right\rangle | z_j \in \mathcal {Z} \right\}$ be two q-ROLSs on $\mathcal {Z}=\left\{ z_1,z_2,\ldots ,z_n \right\}$, $\pounds _{\alpha _1(z_j)}$, $\pounds _{\alpha _2(z_j)}\in \pounds =\left\{ \pounds _0,\pounds _1,\ldots ,\pounds _{2 \tau } \right\}$, and let $g^{*}$ be a linguistic scale function. Further, if the weight of $z_i$ is $\varpi _j\left( \sum _{j=1}^{n}\varpi _j=1 \text { and } 0\le \varpi _j\le 1\right)$, then the novel weighted cosine similarity measure between $\mathcal {Q}_{1}$ and $\mathcal {Q}_{2}$ is denoted and defined as

18 $$\begin{aligned} \breve{\mathcal {C}}_{qlw} \left( \mathcal {Q}_{1},\mathcal {Q}_{2}\right) =\frac{\mathcal {C}_{qlw}\left( \mathcal {Q}_{1},\mathcal {Q}_{2}\right) +1-\mathcal {D}_{qlw}\left( \mathcal {Q}_{1},\mathcal {Q}_{2}\right) }{2}. \end{aligned}$$

Particularly, if $\varpi _j=1\; \forall \; j=1,2,\ldots ,n,$ the presented similarity measure $\breve{\mathcal {C}}_{qlw}$ reduces to the following similarity measure $\breve{\mathcal {C}}_{ql}.$

### **Definition 18**

Let $\mathcal {Q}_{1}=\left\{ \left\langle z_j, \pounds _{\alpha _1(z_j)},\left( \sigma _1(z_j),\varrho _1(z_j) \right) \right\rangle | z_j \in \mathcal {Z} \right\}$ and $\mathcal {Q}_{2}=\left\{ \left\langle z_j, \pounds _{\alpha _2(z_j)},\left( \sigma _2(z_j),\varrho _2(z_j) \right) \right\rangle | z_j \in \mathcal {Z} \right\}$ be two q-ROLSs on $\mathcal {Z}=\left\{ z_1,z_2,\ldots ,z_n \right\}$, $\pounds _{\alpha _1(z_j)}$, $\pounds _{\alpha _2(z_j)}\in \pounds =\left\{ \pounds _0,\pounds _1,\ldots ,\pounds _{2 \tau } \right\}$, and let $g^{*}$ be a linguistic scale function. Then the novel cosine similarity measure between $\mathcal {Q}_{1}$ and $\mathcal {Q}_{2}$ is denoted and defined as

19 $$\begin{aligned} \breve{\mathcal {C}}_{ql} \left( \mathcal {Q}_{1},\mathcal {Q}_{2}\right) =\frac{\mathcal {C}_{ql}\left( \mathcal {Q}_{1},\mathcal {Q}_{2}\right) +1-\mathcal {D}_{ql}\left( \mathcal {Q}_{1},\mathcal {Q}_{2}\right) }{2}. \end{aligned}$$

### **Theorem 6**

*Let* $\mathcal {Q}_{1}$, $\mathcal {Q}_{2}$ and $\mathcal {Q}_{3}$ *be three q-ROLSs. Then the novel weighted cosine similarity measure* $\breve{\mathcal {C}}_{qlw}\left( \mathcal {Q}_{i},\mathcal {Q}_{j}\right) (i,j=1,2,3)$ *meets the following properties*:

$$(p_1)$$.
$$0 \le \breve{\mathcal {C}}_{qlw}\left( \mathcal {Q}_1,\mathcal {Q}_2 \right) \le 1;$$
$$(p_2)$$.
$$\breve{\mathcal {C}}_{qlw}\left( \mathcal {Q}_1,\mathcal {Q}_2 \right) =\breve{\mathcal {C}}_{qlw}\left( \mathcal {Q}_2,\mathcal {Q}_1 \right) ;$$
$$(p_3)$$.
$\breve{\mathcal {C}}_{qlw}\left( \mathcal {Q}_1,\mathcal {Q}_2 \right) =1$ if and only if $\mathcal {Q}_1=\mathcal {Q}_2$, that is, $\alpha _1(z_j)=\alpha _2(z_j)$, $\sigma _1(z_j)=\sigma _2(z_j)$ and $\varrho _1(z_j)=\varrho _2(z_j),$ $\forall \; j=1,2,\ldots ,n.$
$$(p_4)$$.
$$\breve{\mathcal {C}}_{qlw}\left( \mathcal {Q}_1,\mathcal {Q}_3 \right) \le \breve{\mathcal {C}}_{qlw}\left( \mathcal {Q}_1,\mathcal {Q}_2 \right) +\breve{\mathcal {C}}_{qlw}\left( \mathcal {Q}_2,\mathcal {Q}_3 \right) $$ if $$\mathcal {Q}_1\subseteq \mathcal {Q}_2\subseteq \mathcal {Q}_3.$$

### *Proof*

$$(p_1)$$.
According to Theorems 4 and 5, $\mathcal {C}_{qlw}\left( \mathcal {Q}_{1},\mathcal {Q}_{2}\right)$ and $1-\mathcal {D}_{qlw}\left( \mathcal {Q}_{1},\mathcal {Q}_{2}\right) \Rightarrow$
  $0\le \frac{\mathcal {C}_{qlw}\left( \mathcal {Q}_{1},\mathcal {Q}_{2}\right) +1-\mathcal {D}_{qlw}\left( \mathcal {Q}_{1},\mathcal {Q}_{2}\right) }{2}\le 1$. Thus, we get $0 \le \breve{\mathcal {C}}_{qlw}\left( \mathcal {Q}_1,\mathcal {Q}_2 \right) \le 1.$
$$(p_2)$$.
This property is obvious. Therefore, we omit its proof.
$$(p_3)$$.
If $\breve{\mathcal {C}}_{qlw}\left( \mathcal {Q}_1,\mathcal {Q}_2 \right) =1$, according to Eq. (19), we have $\mathcal {C}_{qlw}\left( \mathcal {Q}_{1},\mathcal {Q}_{2}\right) +1-\mathcal {D}_{qlw}\left( \mathcal {Q}_{1},\mathcal {Q}_{2}\right) =2.$ $\mathcal {C}_{qlw}\left( \mathcal {Q}_{1},\mathcal {Q}_{2}\right) -1=\mathcal {D}_{qlw}\left( \mathcal {Q}_{1},\mathcal {Q}_{2}\right) ,$ then $0\le \mathcal {C}_{qlw}\left( \mathcal {Q}_{1},\mathcal {Q}_{2}\right) \left( \mathcal {D}_{qlw}\left( \mathcal {Q}_{1},\mathcal {Q}_{2}\right) \right) \le 1.$ Thus, $\mathcal {C}_{qlw}\left( \mathcal {Q}_{1},\mathcal {Q}_{2}\right) =1$ and $\mathcal {D}_{qlw}\left( \mathcal {Q}_{1},\mathcal {Q}_{2}\right) =0$ are established at the same time. Therefore, according to Theorem 5, $\mathcal {Q}_{1}=\mathcal {Q}_{2}.$ Conversely, if $\mathcal {Q}_{1}=\mathcal {Q}_{2}$, then $\mathcal {C}_{qlw}\left( \mathcal {Q}_{1},\mathcal {Q}_{2}\right) =1$ and $\mathcal {D}_{qlw}\left( \mathcal {Q}_{1},\mathcal {Q}_{2}\right) =0$, so $\breve{\mathcal {C}}_{qlw}\left( \mathcal {Q}_1,\mathcal {Q}_2 \right) =1$ is obtained.
$$(p_4)$$.
Straightforward.

$$\square $$

## Multi-criteria group decision making based on projection method

The present section builds up projection based MCGDM method and discuss its application to EVCS site selection.

### Problem description

In this part, we present q-rung orthopair linguistic approach for MCGDM problems with unknown DMs’ weight. The subsequent mathematical notations are used to solve the q-rung orthopair linguistic MCGDM problems. Let $O=\left\{ o_1,o_2,\ldots ,o_m \right\}$ be a set of *m* available alternatives, and $\wp =\left\{ \wp _1,\wp _2,\ldots ,\wp _n\right\}$ be a set of *n* criteria with weight information $\varpi =\left\{ \varpi _1,\varpi _2,\ldots ,\varpi _n \right\} ,$ where $\varpi _j \in [0,1],\; j=1,2,\ldots ,n,\;$ $\sum _{j=1}^{n}\varpi _j=1$ and a set of *k* DMs $D=\left\{ d_1,d_2,\ldots ,d_k \right\}$. Assume that $\wp _j$ are evaluated for $o_i$ by DM $d_{\ell }$ and depicted as q-rung orthopair linguistic expressions $\mathcal {Q}^{\ell }_{ij}=\left\langle \pounds ^{\ell }_{\alpha _{ij}} ,\left( \sigma ^{\ell }_{ij},\varrho ^{\ell }_{ij}\right) \right\rangle$ $\left( i=1,2,\ldots ,m, j=1,2,\ldots ,n, \ell =1,2,\ldots ,k \right) .$

Afterward, the projection methodology is devised to address the decision making problems with unknown DMs weight information. The detailed calculation procedures are manifested below:

*Step 1*: Establish decision matrices:
  Obtain the linguistic q-rung orthopair fuzzy decision matrix $M^{\ell }$ of the DM $d^{\ell }$.
*Step 2*: Normalization:
  The criteria values in MCGDM problem might be of different types, such as benefit and cost. Since different types of criteria may be neutralized during the aggregation process, it needs to convert different criteria types into the same. Because of cognitive habits, cost-based criteria are typically converted into benefit ones using the following formula:
  20 $$\begin{aligned} \mathcal {Q}^{\ell }_{ij}= {\left\{ \begin{array}{ll} \mathcal {Q}^{\ell }_{ij}, &{} \text{ for } \text{ benefit } \text{ criteria } \wp _j \\ {\left( \mathcal {Q}^{\ell }_{ij}\right) }^c, &{} \text{ for } \text{ cost } \text{ criteria } \wp _j, \end{array}\right. } \end{aligned}$$
  and ${\left( \mathcal {Q}^{\ell }_{ij}\right) }^c$ is the complement of $\mathcal {Q}^{\ell }_{ij}$.
*Step 3*: Collective decision matrix:
  According to the decision matrices $M^{\ell }=\left[ \mathcal {Q}^{\ell }_{ij}\right] _{m\times n}\left( i=1,2,\ldots ,m, j=1,2,\ldots ,n, \ell =1,2,\ldots ,k \right)$ and the weight 1/*k* for all DMs, the collective decision matrix $M=\left[ \mathcal {Q}_{ij}\right] _{m\times n}$ can be yielded by Eq. (21) as follows:
  21 $$\begin{aligned} \mathcal {Q}_{ij}= q-ROLWA \left( \mathcal {Q}^1_{ij},\mathcal {Q}^2_{ij},\ldots ,\mathcal {Q}^k_{ij}\right) =\left\langle \pounds _{\sum _{\ell =1}^{k}}1/k\alpha ^{\ell }_{ij}, \left( \left( 1-\prod ^{k}_{\ell =1}\left( 1-{\left( \sigma ^{\ell }_{ij}\right) }^{q} \right) ^{1/k}\right) ^{1/q},\prod ^{k}_{\ell =1}\left( \varrho ^{\ell }_{ij}\right) ^{1/k} \right) \right\rangle \end{aligned}$$
*Step 4*: Collective similarity measure:
  The collective similarity measure between $M^{\ell } \left( \ell =1,2,\ldots ,k \right)$ and *M* is determined by
  22 $$\begin{aligned} \breve{\mathcal {C}}_{ql}\left( M^{\ell },M \right) =\frac{1}{mn}\sum _{i=1}^{m}\sum _{j=1}^{n}\frac{\mathcal {C}_{ql}\left( \mathcal {Q}^{\ell }_{ij},\mathcal {Q}_{ij}\right) +1-\mathcal {D}_{ql}\left( \mathcal {Q}^{\ell }_{ij},\mathcal {Q}_{ij}\right) }{2} \end{aligned}$$
*Step 5*: DMs weight:
  Determine the weight of DMs by Formula (23):
  23 $$\begin{aligned} \text {w}_{\ell }=\frac{\breve{\mathcal {C}}_{ql}\left( M^{\ell },M \right) }{\sum ^{k}_{\ell =1}\breve{\mathcal {C}}_{ql}\left( M^{\ell },M \right) }, \end{aligned}$$
  where $$0\le \text {w}_{\ell } \le 1,$$ $$\sum ^{k}_{\ell =1}\text {w}_{\ell } =1$$ for $$\ell =1,2,\ldots ,k.$$
*Step 6*: Weighted collective decision matrix:
  According to the derived weight of DMs $\text {w}=\left( \text {w}_{1},\text {w}_{2},\ldots ,\text {w}_{k} \right)$ and the decision matrices $M^{\ell }=\left[ \mathcal {Q}^{\ell }_{ij}\right] _{m\times n}\left( i=1,2,\ldots ,m, j=1,2,\ldots ,n, \ell =1,2,\ldots ,k \right)$, the weighted collective decision matrix $\mathcal {M}=\left[ \mathcal {\check{Q}}_{ij}\right] _{m\times n}$ is obtained as follows:
  24 $$\begin{aligned} \mathcal {\check{Q}}_{ij}= q-ROLWA \left( \mathcal {Q}^1_{ij},\mathcal {Q}^2_{ij},\ldots ,\mathcal {Q}^k_{ij}\right) =\left\langle \pounds _{\sum _{\ell =1}^{k}}\text {w}_{\ell }\alpha ^{\ell }_{ij}, \left( \left( 1-\prod ^{k}_{\ell =1}\left( 1-{\left( \sigma ^{\ell }_{ij}\right) }^{q} \right) ^{\text {w}_{\ell }}\right) ^{1/q},\prod ^{k}_{\ell =1}\left( \varrho ^{\ell }_{ij}\right) ^{\text {w}_{\ell }} \right) \right\rangle \end{aligned}$$
*Step 7*: Optimal solution:
  Employ the score function and accuracy function of q-ROLS to get the optimal solution $\widetilde{\mathcal {Q}}=\left\{ \widetilde{\mathcal {Q}_{1}},\widetilde{\mathcal {Q}_{2}},\ldots , \widetilde{\mathcal {Q}_{n}}\right\}$ under different criteria. Here, $\widetilde{\mathcal {Q}_{j}}=\left\langle \pounds _{\alpha _{j}},\left( \widetilde{\sigma _{j}},\widetilde{\varrho _{j}}\right) \right\rangle =\max _{1\le i\le m}\left\{ \mathcal {\check{Q}}_{ij}\right\}$; $j=1,2,\ldots n.$
*Step 8*: Weighted similarity measure:
  Determine the weighted similarity measure between each alternative and the optimal solution as follows:
  25 $$\begin{aligned} \breve{\mathcal {C}}_{qlw}\left( \check{\mathcal {Q}_{i}},\widetilde{\mathcal {Q}} \right) =\sum _{j=1}^{n}\varpi _{j}\frac{\mathcal {C}_{ql}\left( \check{\mathcal {Q}}_{ij},\widetilde{\mathcal {Q}_{j}}\right) +1-\mathcal {D}_{ql}\left( \check{\mathcal {Q}}_{ij},\widetilde{\mathcal {Q}_{j}}\right) }{2}. \end{aligned}$$
  Here, $\check{\mathcal {Q}_{i}}=\left\{ \check{\mathcal {Q}_{i1}},\check{\mathcal {Q}_{i2}},\ldots ,\check{\mathcal {Q}_{in}} \right\}$(i.e., the *i*th row in the weighted collective decision matrix $\mathcal {M}$ for the alternative $o_i\left( i=1,2,\ldots ,m \right) ,$ and $\varpi _j\left( \sum _{j=1}^{n}\varpi _j=1 \text { and } 0\le \varpi _j\le 1\right)$ represent the weight of criteria $\wp _j$.
*Step 9*: Ranking:
  Rank the alternatives in accordance with their weighted similarity measure $\breve{\mathcal {C}}_{qlw}$. The larger $\breve{\mathcal {C}}_{qlw}$ means the better alternative.

**Figure 1:**
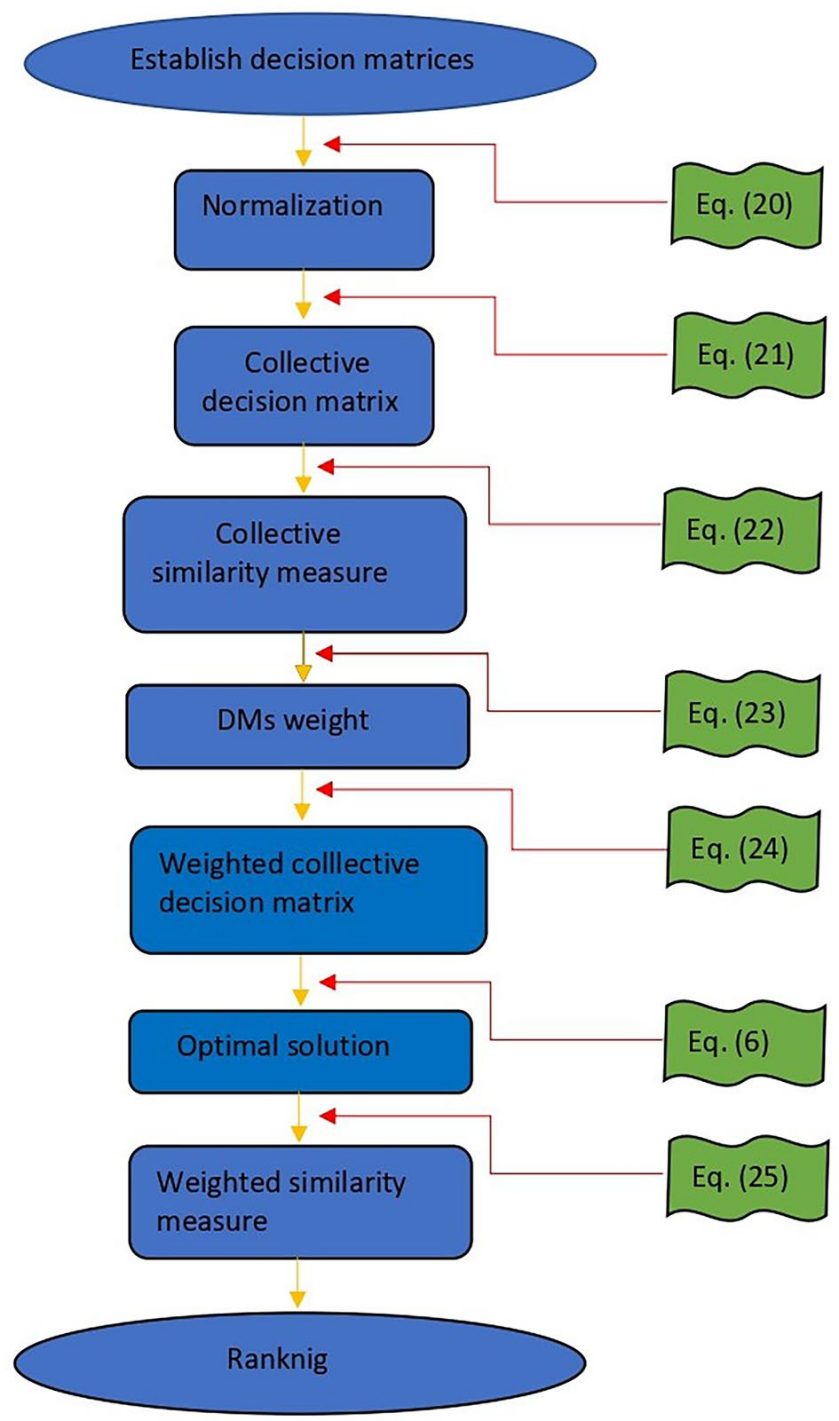
Flow chart of the proposed method.

The flowchart of the presented MCGDM method is depicted in Fig. 1.

### Application

This section is dedicated to present projection-based MCGDM method for q-ROLNs with unknown DMs weights based on stated similarity measures. Furthermore, we apply the proposed technique to electric vehicle charging station (EVCS) site selection and perform in-depth sensitivity analysis to analyze its practicality and stability.

#### Background

In what follows, we briefly describe the considered problem:

Electric vehicles are a type of clean energy transportation that can effectively reduce noise pollution, greenhouse gases, harmful emissions, and fossil energy consumption. With the advancement of battery technology and the worsening of environmental pollution and energy scarcity, electric vehicles are gaining popularity and rapid development worldwide. According to the appropriate development plan carried out by governments and manufacturers, the market share of electric cars would increase in the near future. The large-scale development of electric cars, on the other hand, will create a big gap in charging infrastructure building on the one hand and will pose a threat to the safe and economic functioning of the power grid on the other. The EVCS site selection is seen as an MCGDM problem^47^. As follows, in this part, a numerical scenario for EVCS site selection is developed.

The four underlying EVCS sites $o_i\left( i=1,2,\ldots ,4 \right)$ are to be assessed by using the LTS $\pounds =\left\{ \pounds _{0}= \text {extremely poor},\pounds _{1}=\text { very poor},\pounds _{2}=\text {poor},\pounds _{3}=\text {fair},\pounds _{4}=\text { good},\pounds _{5}=\text { very good},\right. \left. \pounds _{6}=\text { extremely good}\right\}$ under the criteria $\wp _1=$ Traffic convenience, $\wp _2=$ Construction cost (cost criteria), $\wp _3=$ Service capability with weight vector $\left( 0.3,0.3,0.4\right)$ by three DMs as shown in Tables 1, 2 and 3.

**Table 1:** q-rung orthopair linguistic decision matrix provided by $d_{1}$.

	$\wp _1$	$\wp _2$	$\wp _3$
$o_1$	$\left\langle \pounds _{4},\left( 0.8,0.2\right) \right\rangle$	$\left\langle \pounds _{2},\left( 0.5,0.2\right) \right\rangle$	$\left\langle \pounds _{6},\left( 0.4,0.7\right) \right\rangle$
$o_2$	$\left\langle \pounds _{3},\left( 0.3,0.5\right) \right\rangle$	$\left\langle \pounds _{1},\left( 0.7,0.45\right) \right\rangle$	$\left\langle \pounds _{0},\left( 0.5,0.5\right) \right\rangle$
$o_3$	$\left\langle \pounds _{2},\left( 0.3,0.7\right) \right\rangle$	$\left\langle \pounds _{3},\left( 0.8,0.6\right) \right\rangle$	$\left\langle \pounds _{4},\left( 0.4,0.45\right) \right\rangle$
$o_4$	$\left\langle \pounds _{2},\left( 0.55,0.3\right) \right\rangle$	$\left\langle \pounds _{5},\left( 0.35,0.4\right) \right\rangle$	$\left\langle \pounds _{2},\left( 0.7,0.35\right) \right\rangle$

**Table 2:** q-rung orthopair linguistic decision matrix provided by $d_{2}$.

	$\wp _1$	$\wp _2$	$\wp _3$
$o_1$	$\left\langle \pounds _{5},\left( 0.9,0.2\right) \right\rangle$	$\left\langle \pounds _{3},\left( 0.4,0.3\right) \right\rangle$	$\left\langle \pounds _{7},\left( 0.5,0.6\right) \right\rangle$
$o_2$	$\left\langle \pounds _{4},\left( 0.4,0.4\right) \right\rangle$	$\left\langle \pounds _{2},\left( 0.8,0.35\right) \right\rangle$	$\left\langle \pounds _{0},\left( 0.6,0.5\right) \right\rangle$
$o_3$	$\left\langle \pounds _{3},\left( 0.4,0.7\right) \right\rangle$	$\left\langle \pounds _{2},\left( 0.7,0.65\right) \right\rangle$	$\left\langle \pounds _{5},\left( 0.45,0.4\right) \right\rangle$
$o_4$	$\left\langle \pounds _{3},\left( 0.65,0.35\right) \right\rangle$	$\left\langle \pounds _{4},\left( 0.45,0.4\right) \right\rangle$	$\left\langle \pounds _{1},\left( 0.75,0.3\right) \right\rangle$

**Table 3:** q-rung orthopair linguistic decision matrix provided by $d_{3}$.

	$\wp _1$	$\wp _2$	$\wp _3$
$o_1$	$\left\langle \pounds _{3},\left( 0.7,0.25\right) \right\rangle$	$\left\langle \pounds _{1},\left( 0.4,0.3\right) \right\rangle$	$\left\langle \pounds _{5},\left( 0.3,0.75\right) \right\rangle$
$o_2$	$\left\langle \pounds _{2},\left( 0.35,0.5\right) \right\rangle$	$\left\langle \pounds _{0},\left( 0.45,0.7\right) \right\rangle$	$\left\langle \pounds _{1},\left( 0.6,0.5\right) \right\rangle$
$o_3$	$\left\langle \pounds _{1},\left( 0.2,0.7\right) \right\rangle$	$\left\langle \pounds _{2},\left( 0.8,0.6\right) \right\rangle$	$\left\langle \pounds _{4},\left( 0.35,0.55\right) \right\rangle$
$o_4$	$\left\langle \pounds _{3},\left( 0.45,0.4\right) \right\rangle$	$\left\langle \pounds _{6},\left( 0.5,0.5\right) \right\rangle$	$\left\langle \pounds _{3},\left( 0.65,0.5\right) \right\rangle$

#### The decision making process

In this subsection, we employ the suggested technique for the selection of optimal EVCS site selection taking the linguistic scale function $g^*=\frac{\alpha }{2 \tau }$ and $q=2$.

*Step 1* The DMs’ decision matrices are listed in Tables 1, 2 and 3.

*Step 2* Shift the original decision metrics into normalized form (see Tables 4, 5, 6).

**Table 4:** Normalized decision matrix provided by $d_{1}$.

	$\wp _1$	$\wp _2$	$\wp _3$
$o_1$	$\left\langle \pounds _{4},\left( 0.8,0.2\right) \right\rangle$	$\left\langle \pounds _{4},\left( 0.2,0.5\right) \right\rangle$	$\left\langle \pounds _{6},\left( 0.4,0.7\right) \right\rangle$
$o_2$	$\left\langle \pounds _{3},\left( 0.3,0.5\right) \right\rangle$	$\left\langle \pounds _{5},\left( 0.45,0.7\right) \right\rangle$	$\left\langle \pounds _{0},\left( 0.5,0.5\right) \right\rangle$
$o_3$	$\left\langle \pounds _{2},\left( 0.3,0.7\right) \right\rangle$	$\left\langle \pounds _{3},\left( 0.6,0.8\right) \right\rangle$	$\left\langle \pounds _{4},\left( 0.4,0.45\right) \right\rangle$
$o_4$	$\left\langle \pounds _{2},\left( 0.55,0.3\right) \right\rangle$	$\left\langle \pounds _{1},\left( 0.4,0.35\right) \right\rangle$	$\left\langle \pounds _{2},\left( 0.7,0.35\right) \right\rangle$

**Table 5:** Normalized decision matrix provided by $d_{2}$.

	$\wp _1$	$\wp _2$	$\wp _3$
$o_1$	$\left\langle \pounds _{5},\left( 0.9,0.2\right) \right\rangle$	$\left\langle \pounds _{3},\left( 0.3,0.4\right) \right\rangle$	$\left\langle \pounds _{7},\left( 0.5,0.6\right) \right\rangle$
$o_2$	$\left\langle \pounds _{4},\left( 0.4,0.4\right) \right\rangle$	$\left\langle \pounds _{4},\left( 0.35,0.8\right) \right\rangle$	$\left\langle \pounds _{0},\left( 0.6,0.5\right) \right\rangle$
$o_3$	$\left\langle \pounds _{3},\left( 0.4,0.7\right) \right\rangle$	$\left\langle \pounds _{4},\left( 0.65,0.7\right) \right\rangle$	$\left\langle \pounds _{5},\left( 0.45,0.4\right) \right\rangle$
$o_4$	$\left\langle \pounds _{3},\left( 0.65,0.35\right) \right\rangle$	$\left\langle \pounds _{2},\left( 0.4,0.45\right) \right\rangle$	$\left\langle \pounds _{1},\left( 0.75,0.3\right) \right\rangle$

**Table 6:** Normalized decision matrix provided by $d_{3}$.

	$\wp _1$	$\wp _2$	$\wp _3$
$o_1$	$\left\langle \pounds _{3},\left( 0.7,0.25\right) \right\rangle$	$\left\langle \pounds _{5},\left( 0.3,0.4\right) \right\rangle$	$\left\langle \pounds _{5},\left( 0.3,0.75\right) \right\rangle$
$o_2$	$\left\langle \pounds _{2},\left( 0.35,0.5\right) \right\rangle$	$\left\langle \pounds _{6},\left( 0.7,0.45\right) \right\rangle$	$\left\langle \pounds _{1},\left( 0.6,0.5\right) \right\rangle$
$o_3$	$\left\langle \pounds _{1},\left( 0.2,0.7\right) \right\rangle$	$\left\langle \pounds _{4},\left( 0.6,0.8\right) \right\rangle$	$\left\langle \pounds _{4},\left( 0.35,0.55\right) \right\rangle$
$o_4$	$\left\langle \pounds _{3},\left( 0.45,0.4\right) \right\rangle$	$\left\langle \pounds _{0},\left( 0.5,0.5\right) \right\rangle$	$\left\langle \pounds _{3},\left( 0.65,0.5\right) \right\rangle$

*Step 3* Based on Eq. (21), the collective decision matrix is determined as shown in Table 7.

**Table 7:** Collective decision matrix.

	$\wp _1$	$\wp _2$	$\wp _3$
$o_1$	$\left\langle \pounds _{4},\left( 0.8205,0.2154\right) \right\rangle$	$\left\langle \pounds _{4},\left( 0.2713,0.4309\right) \right\rangle$	$\left\langle \pounds _{6},\left( 0.4114,0.6804 \right) \right\rangle$
$o_2$	$\left\langle \pounds _{3},\left( 0.3530,0.4642 \right) \right\rangle$	$\left\langle \pounds _{5},\left( 0.5391,0.6316\right) \right\rangle$	$\left\langle \pounds _{0.3333},\left( 0.5703,0.5000\right) \right\rangle$
$o_3$	$\left\langle \pounds _{2},\left( 0.3131,0.7000 \right) \right\rangle$	$\left\langle \pounds _{3.6667},\left( 0.6177,0.7652\right) \right\rangle$	$\left\langle \pounds _{4.3333},\left( 0.4029,0.4626 \right) \right\rangle$
$o_4$	$\left\langle \pounds _{2.6667},\left( 0.5614,0.3476\right) \right\rangle$	$\left\langle \pounds _{1},\left( 0.4372,0.4286\right) \right\rangle$	$\left\langle \pounds _{2},\left( 0.7035,0.3744 \right) \right\rangle$

*Step 4* In the light of Eq. (22), the collective similarity measure are calculated as $\breve{\mathcal {C}}_{ql}\left( M^{1},M \right) =0.9358,$ $\breve{\mathcal {C}}_{ql}\left( M^{2},M \right) =0.8982,$ $\breve{\mathcal {C}}_{ql}\left( M^{3},M \right) =0.8942.$

*Step 5* According to Eq. (23), the weights of three DMs are $\text {w}_1=0.3430,$ $\text {w}_2=0.3292,$ and $\text {w}_3=0.3278.$

*Step 6* Utilizing Eq. (24), we get the weighted collective decision matrix listed in Table 8.

**Table 8:** Weighted collective decision matrix.

	$\wp _1$	$\wp _2$	$\wp _3$
$o_1$	$\left\langle \pounds _{4.0014},\left( 0.8204,0.2152 \right) \right\rangle$	$\left\langle \pounds _{3.9986},\left( 0.2705,0.4318 \right) \right\rangle$	$\left\langle \pounds _{6.0014},\left( 0.411, 0.6806\right) \right\rangle$
$o_2$	$\left\langle \pounds _{3.0014},\left( 0.3524,0.4646 \right) \right\rangle$	$\left\langle \pounds _{4.9986},\left( 0.5378,0.6328 \right) \right\rangle$	$\left\langle \pounds _{0.3278},\left( 0.5694,0.5000 \right) \right\rangle$
$o_3$	$\left\langle \pounds _{2.0014},\left( 0.3130,0.7000 \right) \right\rangle$	$\left\langle \pounds _{3.6570},\left( 0.6175,0.7656 \right) \right\rangle$	$\left\langle \pounds _{4.3292},\left( 0.4029, 0.4623\right) \right\rangle$
$o_4$	$\left\langle \pounds _{2.6570},\left( 0.5613,0.3468 \right) \right\rangle$	$\left\langle \pounds _{1.0014},\left( 0.4366,0.4273 \right) \right\rangle$	$\left\langle \pounds _{1.9986},\left( 0.7035,0.3739 \right) \right\rangle$

*Step 7* Based on Definition 7, we get the optimal solution $\widetilde{\mathcal {Q}}=\left\{ \left\langle \pounds _{4.0014},\left( 0.8204,0.2152 \right) \right\rangle ,\right. \left. \left\langle \pounds _{4.9986},\left( 0.5378,0.6328 \right) \right\rangle , \left\langle \pounds _{6.0014},\left( 0.411, 0.6806\right) \right\rangle \right\} .$

*Step 8* According to Eq. (25), the weighted similarity measures are $\breve{\mathcal {C}}_{qlw}\left( \check{\mathcal {Q}_{1}},\widetilde{\mathcal {Q}} \right) =0.9457$ $\breve{\mathcal {C}}_{qlw}\left( \check{\mathcal {Q}_{2}},\widetilde{\mathcal {Q}} \right) =0.7327$ $\breve{\mathcal {C}}_{qlw}\left( \check{\mathcal {Q}_{3}},\widetilde{\mathcal {Q}} \right) =0.7376$ $\breve{\mathcal {C}}_{qlw}\left( \check{\mathcal {Q}_{4}},\widetilde{\mathcal {Q}} \right) =0.7067.$

**Figure 2:**
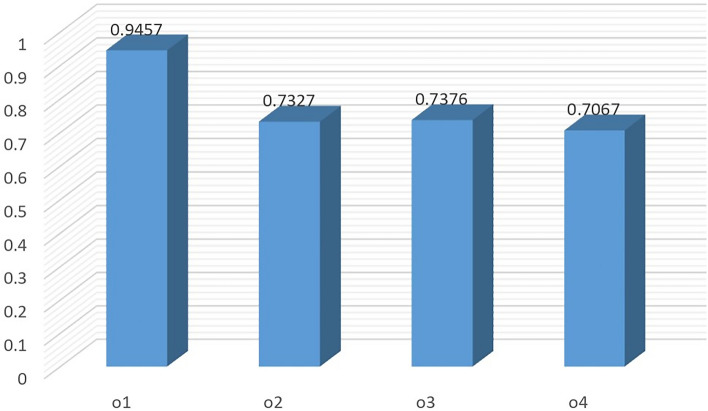
Graphical representation of the proposed method ranking.

*Step 9* Based on Step 8, the ranking result of alternatives is gotten and is shown in Table 13 and Fig. 2.

### Influence of the parameters on the ranking results

In the following subsections, we examine the impact of the parameter *q* and the sensitivity of criteria weights over the ranking orders of considered problem alternatives.

#### Sensitivity analysis with respect to parameter *q*

**Figure 3:**
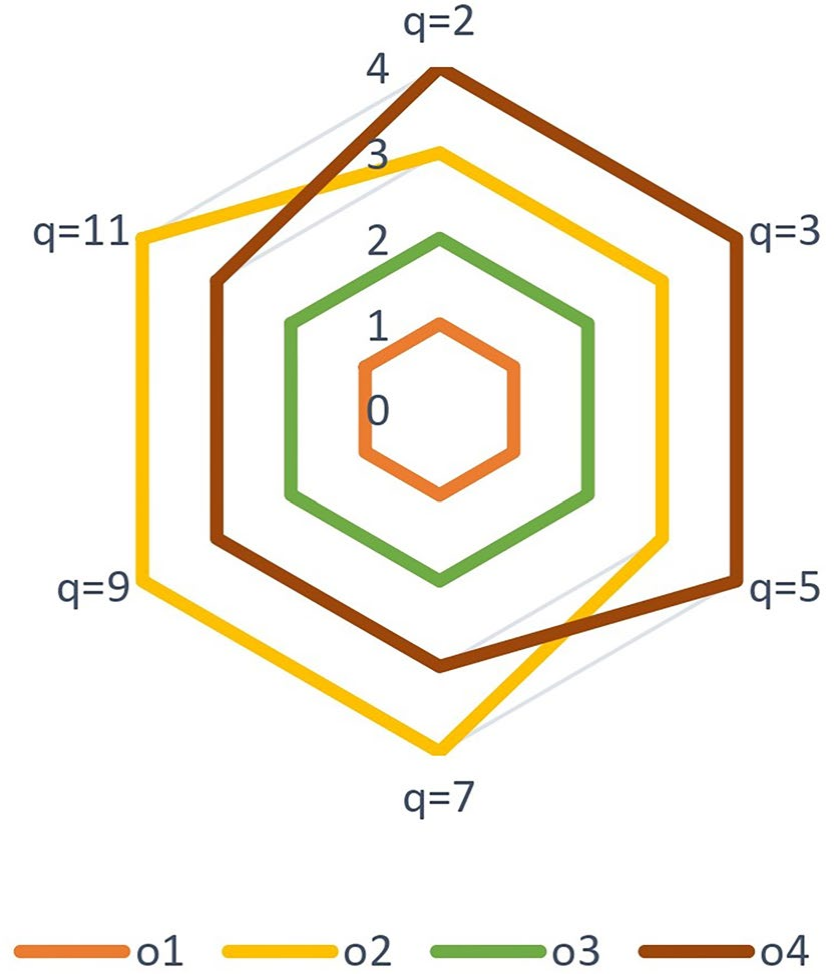
Sensitivity analysis with respect to *q*.

This subsection aims to discuss the impact of the parameter *q* on the ranking orders. The detailed calculation results by altering parameter *q* in the proposed similarity measure are shown in Table 9. The corresponding results with different values of *q* are also manipulated graphically in Fig. 3. From Table 9, we can note that the ranking of alternatives remains stable when $q\in [2,7]$. The ordering positions of $o_2$ and $o_4$ interchange if we set $q\ge 7$. However, the optimal alternative still remains the same, i.e., $o_1$. This indicates that the obtained ordering result of the suggested technique is robust and reliable. In addition, the ranking order of alternative $o_2$ moves down as the value of *q* increases. Thus, the stated method is able to model the degrees of optimism or pessimism displayed by DMs. In general, this method is highly flexible as DMs can choose the value of parameter *q* based on their preferences and theoretical concepts.

**Table 9:** Ranking of alternatives under different values of q.

Parameter	Weighted similarity measures	Ranking
$q=2$	$\breve{\mathcal {C}}_{qlw}\left( \check{\mathcal {Q}_{1}},\widetilde{\mathcal {Q}} \right) =0.9457$, $\breve{\mathcal {C}}_{qlw}\left( \check{\mathcal {Q}_{2}},\widetilde{\mathcal {Q}} \right) = 0.7327$,	$o_1 \succ o_3 \succ o_2 \succ o_4$
	$\breve{\mathcal {C}}_{qlw}\left( \check{\mathcal {Q}_{3}},\widetilde{\mathcal {Q}} \right) =0.7376$, $\breve{\mathcal {C}}_{qlw}\left( \check{\mathcal {Q}_{4}},\widetilde{\mathcal {Q}} \right) =0.7067$	
$q=3$	$\breve{\mathcal {C}}_{qlw}\left( \check{\mathcal {Q}_{1}},\widetilde{\mathcal {Q}} \right) =0.9671$, $\breve{\mathcal {C}}_{qlw}\left( \check{\mathcal {Q}_{2}},\widetilde{\mathcal {Q}} \right) = 0.6999$,	$o_1 \succ o_3 \succ o_2 \succ o_4$
	$\breve{\mathcal {C}}_{qlw}\left( \check{\mathcal {Q}_{3}},\widetilde{\mathcal {Q}} \right) = 0.7251$, $\breve{\mathcal {C}}_{qlw}\left( \check{\mathcal {Q}_{4}},\widetilde{\mathcal {Q}} \right) =0.6790$	
$q=5$	$\breve{\mathcal {C}}_{qlw}\left( \check{\mathcal {Q}_{1}},\widetilde{\mathcal {Q}} \right) =0.9669$, $\breve{\mathcal {C}}_{qlw}\left( \check{\mathcal {Q}_{2}},\widetilde{\mathcal {Q}} \right) = 0.6509$,	$o_1 \succ o_3 \succ o_2 \succ o_4$
	$\breve{\mathcal {C}}_{qlw}\left( \check{\mathcal {Q}_{3}},\widetilde{\mathcal {Q}} \right) =0.7287$, $\breve{\mathcal {C}}_{qlw}\left( \check{\mathcal {Q}_{4}},\widetilde{\mathcal {Q}} \right) =0.6477$	
$q=7$	$\breve{\mathcal {C}}_{qlw}\left( \check{\mathcal {Q}_{1}},\widetilde{\mathcal {Q}} \right) = 0.9612$, $\breve{\mathcal {C}}_{qlw}\left( \check{\mathcal {Q}_{2}},\widetilde{\mathcal {Q}} \right) =0.6170$	$o_1 \succ o_3 \succ o_4 \succ o_2$
	$\breve{\mathcal {C}}_{qlw}\left( \check{\mathcal {Q}_{3}},\widetilde{\mathcal {Q}} \right) =0.7328$, $\breve{\mathcal {C}}_{qlw}\left( \check{\mathcal {Q}_{4}},\widetilde{\mathcal {Q}} \right) =0.6384$	
$q=9$	$\breve{\mathcal {C}}_{qlw}\left( \check{\mathcal {Q}_{1}},\widetilde{\mathcal {Q}} \right) =0.9526$, $\breve{\mathcal {C}}_{qlw}\left( \check{\mathcal {Q}_{2}},\widetilde{\mathcal {Q}} \right) =0.5920$,	$o_1 \succ o_3 \succ o_4 \succ o_2$
	$\breve{\mathcal {C}}_{qlw}\left( \check{\mathcal {Q}_{3}},\widetilde{\mathcal {Q}} \right) =0.7302$, $\breve{\mathcal {C}}_{qlw}\left( \check{\mathcal {Q}_{4}},\widetilde{\mathcal {Q}} \right) =0.6359$	
$q=11$	$\breve{\mathcal {C}}_{qlw}\left( \check{\mathcal {Q}_{3}},\widetilde{\mathcal {Q}} \right) = 0.9423$, $\breve{\mathcal {C}}_{qlw}\left( \check{\mathcal {Q}_{4}},\widetilde{\mathcal {Q}} \right) =0.5735$	$o_1 \succ o_3 \succ o_4 \succ o_2$
	$\breve{\mathcal {C}}_{qlw}\left( \check{\mathcal {Q}_{4}},\widetilde{\mathcal {Q}} \right) =0.7227$ $\breve{\mathcal {C}}_{qlw}\left( \check{\mathcal {Q}_{4}},\widetilde{\mathcal {Q}} \right) =0.6356$	

#### Sensitivity analysis with respect to criteria weight

**Figure 4:**
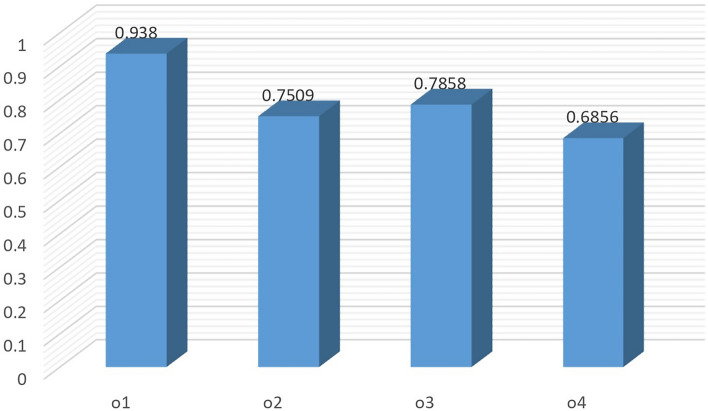
Ranking results when $\varpi _1=0.2$, $\varpi _2=0.343$ and $\varpi _3=0.457$.

This section concentrates on sensitivity analysis with respect to criteria weight. In the criteria weight analysis, the weights of the given criteria are assigned by changing the weight of each criteria proportionally to the weights of other criteria. In this article, we have taken the weights $\varpi _1=0.3$, $\varpi _2=0.3$ and $\varpi _3=0.4$ for $\wp _1$, $\wp _2$ and $\wp _3$, respectively. Now, if we change the weight $\varpi _1$ from 0.3 to 0.2. The weights of other criteria, i.e., $\varpi _2$ and $\varpi _3$ becomes 0.343 and 0.457. These weights are determined as follows: By taking $\varpi _1=0.2$, the sum of $\varpi _2$ and $\varpi _3$ becomes $1-0.2=0.8$. The sum of these weights is used to compute the ratio of the weights for the remaining criteria. The weight of $\wp _3$ is $\varpi _3=\frac{0.4}{1-0.3}\times 0.8=0.457$. Analogously, the weight of $\wp _2$ is 0.343. The ranking results are recalculated by taking these new weights of criteria. The obtained results for this analysis are shown in Fig. 4 and Table 10. From the results, it is clear that there is no sensitivity, i.e., the proposed approach is stable against the slight changes in the criteria weights.

**Figure 5:**
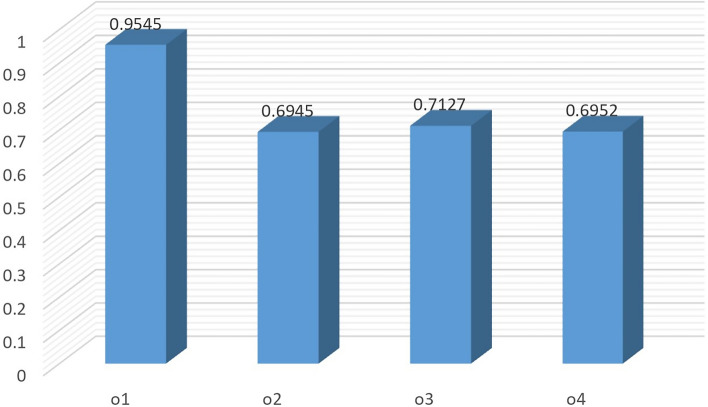
Ranking results when $\varpi _1=0.343$, $\varpi _2=0.2$ and $\varpi _3=0.457$.

By taking $\varpi _2=0.2$, we get $\varpi _1=0.343$ and $\varpi _3=0.457$. The resulting ranking is shown in Fig. 5. In this case the alternatives $o_2$ and $o_4$ have replaced their positions.

**Figure 6:**
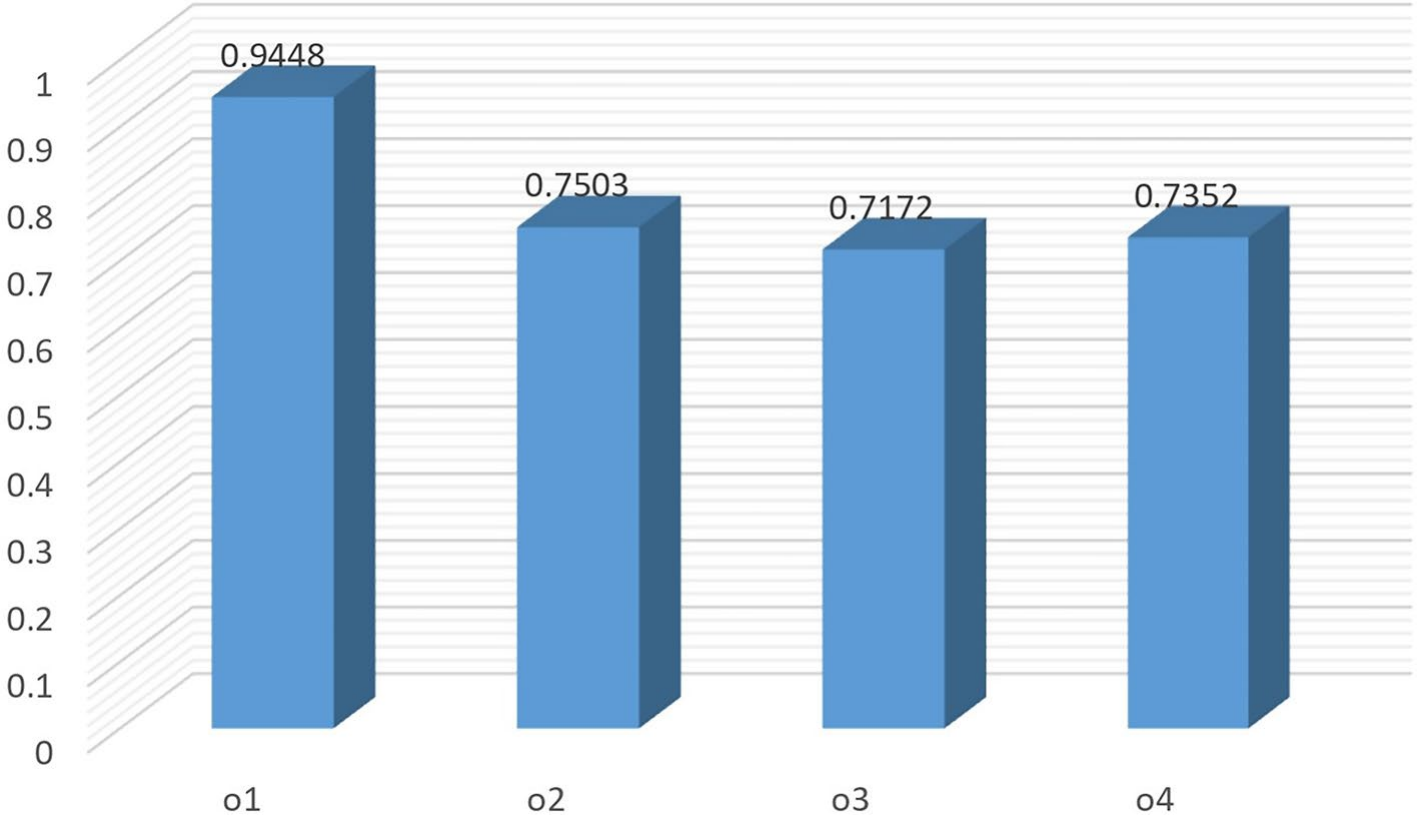
Ranking results when $\varpi _1=0.35$, $\varpi _2=0.35$ and $\varpi _3=0.3$.

If we reduce the weight of criteria $\wp _3$ to 0.3. This switches the weights of $\wp _1$ and $\wp _2$ to 0.35 and 0.35, respectively. The ranking of alternatives for these changing to weight values is sensitive, as can be seen from Table 10. Only the most preferable alternative has maintained its position while the remaining alternatives have altered their ordering positions (see Fig. 6).

**Table 10:** Ranking of alternatives under different values of criteria.

Criteria weights	Weighted similarity measures	Ranking
$\varpi _1=0.300,$ $\varpi _2=0.300,$	$\breve{\mathcal {C}}_{qlw}\left( \check{\mathcal {Q}_{1}},\widetilde{\mathcal {Q}} \right) =0.9457$, $\breve{\mathcal {C}}_{qlw}\left( \check{\mathcal {Q}_{2}},\widetilde{\mathcal {Q}} \right) = 0.7327$,	
$\varpi _3=0.400$	$\breve{\mathcal {C}}_{qlw}\left( \check{\mathcal {Q}_{3}},\widetilde{\mathcal {Q}} \right) =0.7376$, $\breve{\mathcal {C}}_{qlw}\left( \check{\mathcal {Q}_{4}},\widetilde{\mathcal {Q}} \right) =0.7067$	$o_1 \succ o_3 \succ o_2 \succ o_4$
$\varpi _1=0.200,$ $\varpi _2=0.343,$	$\breve{\mathcal {C}}_{qlw}\left( \check{\mathcal {Q}_{1}},\widetilde{\mathcal {Q}} \right) =0.9380$, $\breve{\mathcal {C}}_{qlw}\left( \check{\mathcal {Q}_{2}},\widetilde{\mathcal {Q}} \right) = 0.7509$,	
$\varpi _3=0.457$	$\breve{\mathcal {C}}_{qlw}\left( \check{\mathcal {Q}_{3}},\widetilde{\mathcal {Q}} \right) =0.7858$, $\breve{\mathcal {C}}_{qlw}\left( \check{\mathcal {Q}_{4}},\widetilde{\mathcal {Q}} \right) =0.6856$	$o_1 \succ o_3 \succ o_2 \succ o_4$
$\varpi _1=0.343,$ $\varpi _2=0.200,$	$\breve{\mathcal {C}}_{qlw}\left( \check{\mathcal {Q}_{1}},\widetilde{\mathcal {Q}} \right) = 0.9545$, $\breve{\mathcal {C}}_{qlw}\left( \check{\mathcal {Q}_{2}},\widetilde{\mathcal {Q}} \right) = 0.6945$,	
$\varpi _3=0.457$	$\breve{\mathcal {C}}_{qlw}\left( \check{\mathcal {Q}_{3}},\widetilde{\mathcal {Q}} \right) = 0.7127$, $\breve{\mathcal {C}}_{qlw}\left( \check{\mathcal {Q}_{4}},\widetilde{\mathcal {Q}} \right) =0.6952$	$o_1 \succ o_3 \succ o_4 \succ o_2$
$\varpi _1=0.350,$ $\varpi _2=0.350,$	$\breve{\mathcal {C}}_{qlw}\left( \check{\mathcal {Q}_{1}},\widetilde{\mathcal {Q}} \right) =0.9448$, $\breve{\mathcal {C}}_{qlw}\left( \check{\mathcal {Q}_{2}},\widetilde{\mathcal {Q}} \right) = 0.7503$,	
$\varpi _3=0.300$	$\breve{\mathcal {C}}_{qlw}\left( \check{\mathcal {Q}_{3}},\widetilde{\mathcal {Q}} \right) =0.7172$, $\breve{\mathcal {C}}_{qlw}\left( \check{\mathcal {Q}_{4}},\widetilde{\mathcal {Q}} \right) =0.7352$	$o_1 \succ o_2 \succ o_4 \succ o_3$

### Validity test for the proposed method

Motivated by the literature^48,49^, this analysis established the presented method’s validity. The preferred relationship among alternatives satisfies the following three criteria:

1. *Non-substitutability*: When a non-optimal alternative is replaced with a worse alternative, an effective MCGDM approach should not modify the indication of the best alternative without altering the relative weight of any decision criteria^49^.
2. *Transitivity*: An effective approach should adhere to the transitive property. That is, if $o_i\succ o_k$ and $o_k\succ o_l$, then $o_i\succ o_l$, $\left( i,k,l=1,2,\ldots ,m\right) .$
3. *Consistency*: When all alternatives are treated as a set and each subset of the set is ranked using the same evaluation technique, the preference relations between the subset’s alternatives and the set’s alternatives are consistent. That is, there is a set of alternatives $O=\left\{ o_i,o_j,o_k,o_l \right\}$, and MCGDM approach produces the following sorting result: $o_i\succ o_j\succ o_k \succ o_l$. If $\acute{O}=\left\{ o_i,o_k,o_l \right\}$ is a subset of *O*, then the outcome of sorting is $o_i\succ o_k \succ o_l$.

These criteria are tested on the suggested approach as follows:

#### Criteria 1

If we choose $o_2$ as the non-optimal alternative and $o_4$ as the worse alternative, and switch their rating values in Tables 1, 2 and 3. Then, employing the proposed approach, we get the weighted similarity measure of each alternative $o_i$ as: $\breve{\mathcal {C}}_{qlw}\left( \check{\mathcal {Q}_{1}},\widetilde{\mathcal {Q}} \right) =0.9265$, $\breve{\mathcal {C}}_{qlw}\left( \check{\mathcal {Q}_{2}},\widetilde{\mathcal {Q}} \right) =0.6972$, $\breve{\mathcal {C}}_{qlw}\left( \check{\mathcal {Q}_{3}},\widetilde{\mathcal {Q}} \right) =0.7434$ and $\breve{\mathcal {C}}_{qlw}\left( \check{\mathcal {Q}_{4}},\widetilde{\mathcal {Q}} \right) =0.7223$. Hence, ordering position of alternatives becomes: $o_1\succ o_3 \succ o_4 \succ o_2.$ Because the best alternative is $o_1$, which is the same as it was before switching data, it is proven that the suggested technique does not modify the indication of the best alternative when a non-optimal alternative is substituted with another worst alternative. Thus, the suggested technique is valid for the non-substitutability of criteria 1^49^.

#### Criteria 2 and 3

In these two criteria, we split the original MCGDM problem into three smaller ones, which consist of alternatives $\left\{ o_1,o_2,o_3 \right\}$, $\left\{ o_2,o_3,o_4 \right\}$ and $\left\{ o_3,o_4,o_1 \right\}$. Based on this, we employ the suggested technique to each sub-problem, and the corresponding ranking orders are $o_1\succ o_3\succ o_2,$ $o_3\succ o_2 \succ o_1$ and $o_1\succ o_3 \succ o_4,$ respectively. By combining these orderings, we obtain $o_1\succ o_3 \succ o_2 \succ o_4$ , demonstrating that the transitivity condition holds and the final ranking is equal to the un-decomposed problem. As a result, we conclude that the suggested approach is valid for the transitivity of criteria 2 and consistency of criteria 3.

## Comparative study

To further demonstrate the validity and supremacy of the presented MCGDM approach, we utilize the other three existing approaches to address the same EVCS selection problem delineated in “Multi-criteria group decision making based on projection method” section. These existing MCGDM methods include WHM and WGHM operators-based method^30^, DSET-based method with ILNs^28^, and q-rung orthopair weighted cosine similarity measures approach^22^.

### Heronian operators based method

In the first phase, we make a comparison with Li et al.^30^ work. The computational steps and results are listed as follows:

**Table 11:** WHM Collective decision matrix.

	$\wp _1$	$\wp _2$	$\wp _3$
$o_1$	$\left\langle \pounds _{4.0221},\left( 0.8146,0.2164\right) \right\rangle$	$\left\langle \pounds _{4.0187},\left( 0.2759,0.4331\right) \right\rangle$	$\left\langle \pounds _{6.0155},\left( 0.4127,0.6825\right) \right\rangle$
$o_2$	$\left\langle \pounds _{3.0287},\left( 0.3529,0.4663\right) \right\rangle$	$\left\langle \pounds _{5.0147},\left( 0.5363 ,0.6447\right) \right\rangle$	$\left\langle \pounds _{0.4015},\left( 0.5692,0.5000\right) \right\rangle$
$o_3$	$\left\langle \pounds _{2.0419},\left( 0.3160,0.7000\right) \right\rangle$	$\left\langle \pounds _{3.6626},\left( 0.6174,0.7733\right) \right\rangle$	$\left\langle \pounds _{4.3347},\left( 0.4033,0.4654\right) \right\rangle$
$o_4$	$\left\langle \pounds _{2.6654},\left( 0.5606 ,0.3493\right) \right\rangle$	$\left\langle \pounds _{1.0796},\left( 0.4369,0.4319\right) \right\rangle$	$\left\langle \pounds _{2.0385},\left( 0.7028,0.3805\right) \right\rangle$

**Table 12:** WGHM Collective decision matrix.

	$\wp _1$	$\wp _2$	$\wp _3$
$o_1$	$\left\langle \pounds _{3.9505},\left( 0.7977,0.2183\right) \right\rangle$	$\left\langle \pounds _{3.9463},\left( 0.2657,0.4386\right) \right\rangle$	$\left\langle \pounds _{5.9695},\left( 0.3978,0.6895\right) \right\rangle$
$o_2$	$\left\langle \pounds _{2.9312},\left( 0.3494 ,0.4708\right) \right\rangle$	$\left\langle \pounds _{4.9574},\left( 0.4925 ,0.6825\right) \right\rangle$	$\left\langle \pounds _{0},\left( 0.5657,0.4999\right) \right\rangle$
$o_3$	$\left\langle \pounds _{1.8890},\left( 0.2967 ,0.6999\right) \right\rangle$	$\left\langle \pounds _{3.5441},\left( 0.6164,0.7708\right) \right\rangle$	$\left\langle \pounds _{4.3122},\left( 0.3995,0.4731\right) \right\rangle$
$o_4$	$\left\langle \pounds _{2.6253},\left( 0.5484 ,0.3528\right) \right\rangle$	$\left\langle \pounds _{0},\left( 0.4325,0.4390\right) \right\rangle$	$\left\langle \pounds _{1.8856},\left( 0.6997 ,0.3973\right) \right\rangle$

*Step 1*: Likewise the proposed approach, we first normalize the provided decision matrices, which have already been normalized in “Multi-criteria group decision making based on projection method” section as shown in Tables 4, 5 and 6.
*Step 2*: Secondly, the weighted collective decision matrix is obtained according to WHM or WGHM operator. The derived collective decision matrices by WHM and WGHM operators are listed in Table 11 and 12, respectively.
*Step 3*: Thirdly, the aggregated values of alternatives are computed by utilizing these operators as follows.
  Aggregated values of alternatives produced by WHM operator: $o_1=\left\langle \pounds _{4.8926},\left( 0.5823,0.4430\right) \right\rangle$, $o_2=\left\langle \pounds _{2.7025},\left( 0.5079,0.5348\right) \right\rangle$,
  $$o_3=\left\langle \pounds _{3.5134},\left( 0.4685,0.6308\right) \right\rangle $$, $$o_4=\left\langle \pounds _{1.9689},\left( 0.5977,0.3891\right) \right\rangle $$.
  Aggregated values of alternatives produced by WGHM operator:
  $$o_1=\left\langle \pounds _{5.9989},\left( 0.4612 ,0.5333\right) \right\rangle $$, $$o_2=\left\langle \pounds _{0},\left( 0.4729 ,0.5603\right) \right\rangle $$,
  $$o_3=\left\langle \pounds _{3.3097},\left( 0.4303,0.6582\right) \right\rangle $$, $$o_4=\left\langle \pounds _{0},\left( 0.5668,0.3988\right) \right\rangle $$.
*Step 4*: Based on Definition 7, the score value of each alternative is computed as detailed below:
  Score values of alternatives produced by WHM operator:
  $$S(o_1)=5.5741,$$ $$S(o_2)=2.6298,$$ $$S(o_3)=2.943,$$ $$S(o_4)=2.3796.$$
  Score values of alternatives produced by WGHM operator:
  $$S(o_1)=5.5664,$$ $$S(o_2)=0,$$ $$S(o_3)=2.5554,$$ $$S(o_4)=0.$$
*Step 5*: Finally, rank the alternatives accordingly to their score values in descending order. The obtained ranking results for WHM and WGHM are tabulated in Table 13.

It is evident from Table 13 that the ranking results obtained by the WHM operator is identical to the ranking obtained by our constructed method. Obviously, this verifies the validity of the suggested approach. Apart from the WHM operator, we can observe from Table 13 that the preferred positions of the alternatives $o_1$ and $o_3$ produced by the WGHM operator also match with our derived results. But the alternatives $o_2$ and $o_4$ are incomparable because the score and accuracy values of these two alternatives are the same. Thus, the ranking result produced by the WGHM operator is unreasonable. It is worth noting that whenever there is a linguistic term $\pounds _{0}$ in q-ROLN, WGHM leads to unreasonable results. Let us illustrate this defect by Example 5.

#### *Example 5*

Let $\mathcal {Q}_{1}=\left\langle \pounds _{\alpha _1},\left( \sigma _1,\varrho _1 \right) \right\rangle$,and $\mathcal {Q}_{2}= \left\langle \pounds _{0},\left( \sigma _2,\varrho _2 \right) \right\rangle$ be two q-ROLNs, if the multiplicative operation $\otimes$ defined in Definition 9 is performed on the q-ROLNs $\mathcal {Q}_{1}$ and $\mathcal {Q}_{2}$, namely, $\mathcal {Q}_{1}\otimes \mathcal {Q}_{2}$, then the linguistic part result is always $\pounds _{0}$ no matter what the value of the linguistic part of $\mathcal {Q}_{1}$ is.

From the above example, it is clear that the WGHM operator is invalid when the linguistic term of any q-ROLN is $\pounds _{0}$. Since the WGHM operator is based on the multiplication operation, we get the same results in this situation. However, in practice, it is often unavoidable that the linguistic term is $\pounds _{0}$ in q-ROLNs. In such situation, the WGHM operator is not capable to obtain fruitful results.

### DSET-based method

This part is devoted to address the considered EVCS selection problem through Liu and Zhang approach^28^. But this study is based on intuitionstic linguistic information. From Table 4, we can observe that in the assessment information, most of the data membership and no-membership grades sum is greater than one. For instance, $\left\langle \pounds _{6},\left( 0.4,0.7\right) \right\rangle$, we have $0.4+0.7\nleq 1$. Now, by allowing such information in the evaluation matrices Liu and Zhang approach is not capable to solve the problem. Thus, the existing method^28^ has a lack of fuzzy information and is not capable to address complex scenario problems. Basically, $\left\langle \pounds _{6},\left( 0.4,0.7\right) \right\rangle$ is q-ROLN and^28^ is valid only for ILNs. q-ROLNs are clearly better than ILNs since they can represent evaluation criterion values of alternatives more comprehensively. By using a parameter q, q-ROLNs are more adaptable in expressing fuzzy information. As the value of the parameter q increases, the breadth of the fuzzy information expands. This analysis shows that the stated MCGDM method is superior and more capable than the available methods.

**Table 13:** Ranking results from different methods.

Method	Ranking
$(r_1)$–WHM operator^30^	$o_1\succ o_3 \succ o_2 \succ o_4$
$(r_2)$–WGHM operator^30^	$o_1\succ o_3 \succ o_2= o_4$
$(r_3)$–Liu and Zhang approach^28^	Not applicable
$(r_4)$–Cosine similarity measure $\breve{\mathcal {C}}_{qlw1}$^22^	$o_1\succ o_4 \succ o_2\succ o_3$
$(r_5)$–Cosine similarity measure $\breve{\mathcal {C}}_{qlw2}$^22^	$o_1\succ o_2 \succ o_4\succ o_3$
$(r_6)$–Proposed method	$o_1\succ o_3 \succ o_2 \succ o_4$

### Cosine similarity measures approach with q-ROFNs

q-rung orthopair cosine similarity measures regards only the q-rung orthopair fuzzy information as the belief degrees of alternatives on criteria regarding membership and non-membership grades based on the known weight vector of criteria. In what follows, we use these measures in the proposed approach to determine the degree of similarity between each alternative and optimal solution and rank the alternatives.

Because the similarity measures presented by^22^ can only tackle the problems with q-rung orthhopair fuzzy information. Thereby, we first reduce the decision matrix (depicted in Table 8) and optimal solution derived in Step 7, into q-ROFNs by removing the linguistic parts from q-ROLNs. The deduced weighted collective decision matrix is outlined in Table 14, while the optimal solution is $\widetilde{\mathcal {Q}}=\left\{ \left\langle 0.8204,0.2152 \right\rangle , \left\langle 0.5378,0.6328 \right\rangle , \left\langle 0.411, 0.6806 \right\rangle \right\}$.

**Table 14:** Weighted collective q-rung orthopair fuzzy decision matrix.

	$\wp _1$	$\wp _2$	$\wp _3$
$o_1$	$\left\langle 0.8204,0.2152 \right\rangle$	$\left\langle 0.2705,0.4318 \right\rangle$	$\left\langle 0.411, 0.6806 \right\rangle$
$o_2$	$\left\langle 0.3524,0.4646 \right\rangle$	$\left\langle 0.5378,0.6328\right\rangle$	$\left\langle 0.5694,0.5000 \right\rangle$
$o_3$	$\left\langle 0.3130,0.7000 \right\rangle$	$\left\langle 0.6175,0.7656 \right\rangle$	$\left\langle 0.4029, 0.4623 \right\rangle$
$o_4$	$\left\langle 0.5613,0.3468 \right\rangle$	$\left\langle 0.4366,0.4273 \right\rangle$	$\left\langle 0.7035,0.3739 \right\rangle$

Then the cosine similarity measures $\breve{\mathcal {C}}_{qlw1}$ and $\breve{\mathcal {C}}_{qlw2}$^22^ of alternatives can be gotten as follows:

Weighted similarity measures are $\breve{\mathcal {C}}_{qlw1}\left( \check{\mathcal {A}_{1}},\widetilde{\mathcal {A}} \right) =0.9906$, $\breve{\mathcal {C}}_{qlw1}\left( \check{\mathcal {A}_{2}},\widetilde{\mathcal {A}} \right) =0.8051$,

$$\breve{\mathcal {C}}_{qlw1}\left( \check{\mathcal {A}_{3}},\widetilde{\mathcal {A}} \right) =0.7607$$, $$\breve{\mathcal {C}}_{qlw1}\left( \check{\mathcal {A}_{4}},\widetilde{\mathcal {A}} \right) =0.8161$$.

**Figure 7:**
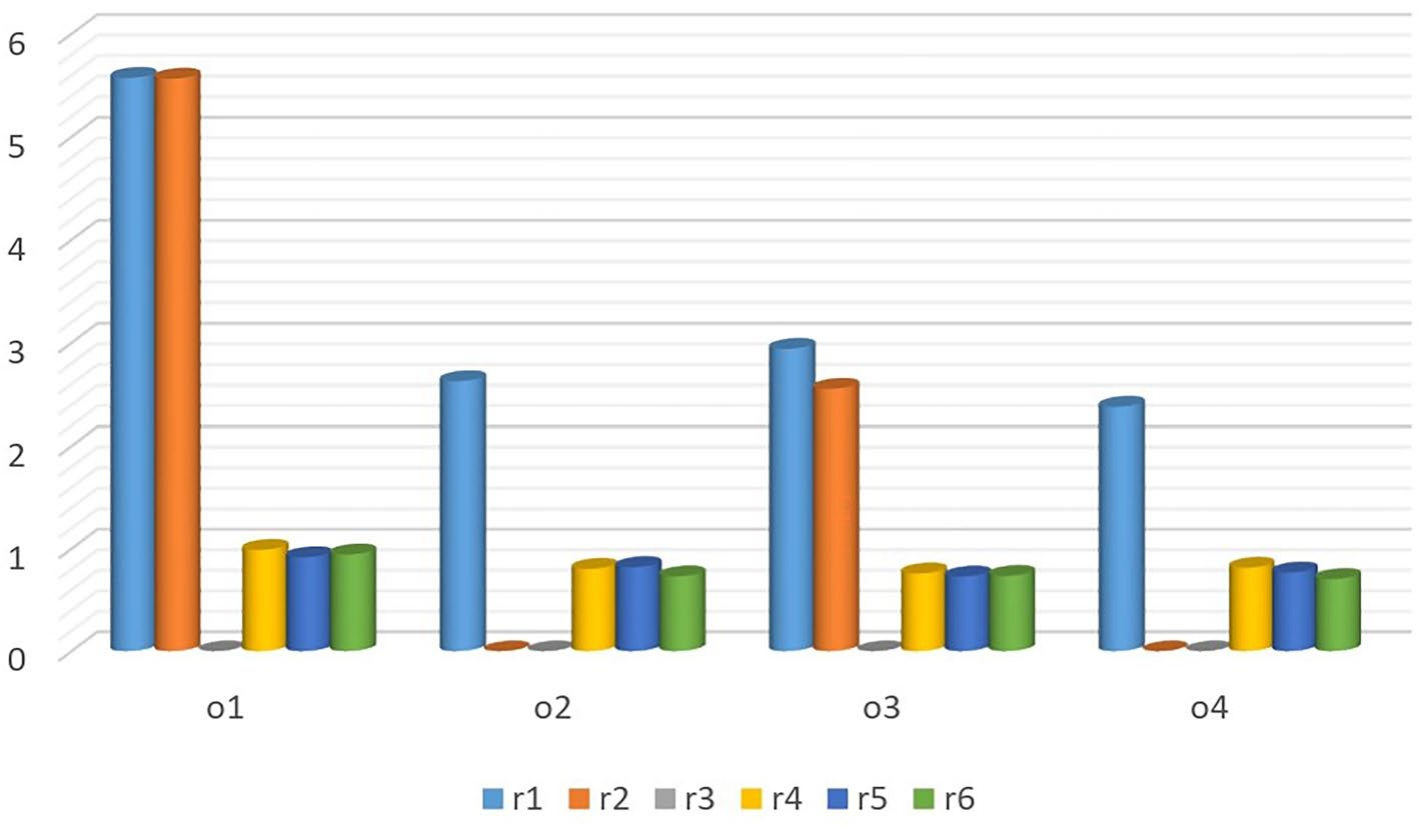
Ranking of EVCS based on different methods.

Weighted similarity measures are $\breve{\mathcal {C}}_{qlw2}\left( \check{\mathcal {A}_{1}},\widetilde{\mathcal {A}} \right) =0.9183$, $\breve{\mathcal {C}}_{qlw2}\left( \check{\mathcal {A}_{2}},\widetilde{\mathcal {A}} \right) =0.8225$,

$$\breve{\mathcal {C}}_{qlw2}\left( \check{\mathcal {A}_{3}},\widetilde{\mathcal {A}} \right) =0.7311$$, $$\breve{\mathcal {C}}_{qlw2}\left( \check{\mathcal {A}_{4}},\widetilde{\mathcal {A}} \right) =0.7703$$.

and finally, the ranking of EVCS sites is obtained as shown in Table 13 and Fig. 7.

From Table 13, we can observe that the results obtained by q-rung orthopair fuzzy cosine measures are entirely different from the suggested technique’s outcomes. This is due to the fact that Liu and Zhang approach^28^ is based on q-ROFNs and is ineffective when dealing with MCGDM problems with q-ROLNs. To solve the considered problem using this technique, we discard the LTS from q-ROLNs, resulting in a significant loss of original assessment information. As a result, an irrational ranking is obtained.

The main merits of the presented method in comparison to other methods are listed in the following.

(i).: Unlike Li et al. approach^30^, the proposed method is more appropriate for practical problems. For example, when there is a linguistic term of one criteria is equal to $\pounds _{0}$, the scoring function of the corresponding alternative will result in zero. Thus, it leads to biased results (see Table 13). On the other hand, the developed method has overcome this defect by using the proposed cosine measures.
(ii).: Unlike the existing q-rung orthopair linguistic methods, the proposed approach has the capability to determine the unknown weights of DMs.
(iii).: The method adopted here aims to find the best alternative through the proposed regular similarity measure, while the previous similarity measures^22^ do not satisfy the axiom of regularity. For that reason, the evaluation results obtained by utilizing the proposed measures are more evident and easy to distinguish in comparison to^22^.
(iv).: Compared with the existing method established in intuitionistic linguistic environment^28^, our provided method is based on q-ROLNs which have a wide range to describe real cases.

In addition to the comparative analysis presented above, we provide some characteristic comparisons of our proposed MCGDM method with the prevailing approaches in Table 15.

**Table 15:** Characteristic comparison of different approaches.

	Aggregation-based method^30^	DSET-based method^28^	Similarity measure-based method^22^	Proposed method
Data	Both qualitative and quantitative	Only qualitative	Only quantitative	Both qualitative and quantitative
Range of information	Wider	Limited	Wider	Wider
Weights	Known	Known	Known	Unknown
Extra parameter	Yes	No	Yes	Yes

## Conclusions and ongoing work

q-ROLS is a hybrid mathematical tool, which combines the advantages of q-ROFS and LTS has caught the attention of scholars. In this work, certain additional operational laws and relations with some results are originated as a useful supplement to the existing operational laws of q-ROLSs. Keeping the necessity of similarity measures, several q-rung orthopair linguistic cosine and weighted cosine similarity measures are investigated based on the linguistic scale function to consider the semantics of linguistic terms. The prominent characteristic of these proposed similarity measures is that they fulfill the required properties of the regular similarity measure. Afterward, we pioneered the MCGDM method with q-ROLNs and unknown weights of DMs. We further applied the stated approach to MCGDM problem concerning EVCS site selection to illustrate its implementation. Sensitivity analysis with respect to criteria weight and parameter *q* is carried out to light on the stability. The validity test was also performed. Meanwhile, the derived results were compared with prevailing techniques to show the effectiveness and supremacies of the proposed method. The results for this analysis were shown in tabular and graphical representation.

In ongoing research, we aim to discuss the following topics:

(1).: The improvement of the existing operational laws of q-ROLNs and their utilization in aggregation operators such as Bonferroni mean operator and Maclaurin symmetric mean operator.
(2).: The introduction of a generalized q-rung orthopair linguistic soft set to expand the implantation scope of q-ROLS.
(3).: The expansion of the developed approach to some other fuzzy environments such as T-spherical fuzzy environments and interval-valued T-spherical fuzzy environments where DMs have a variety of choices for the selection of membership, non-membership, and neutral grades.
(4).: The investigation of q-rung orthopair linguistic optimization model for fully and partially unknown criteria weight determination.

## Data Availability

The datasets used and/or analysed during the current study available from the corresponding author on reasonable request.
